# What is really out there? Review of the genus *Okenia* Menke, 1830 (Nudibranchia: Goniodorididae) in the Mediterranean Sea with description of two new species

**DOI:** 10.1371/journal.pone.0215037

**Published:** 2019-05-01

**Authors:** Marta Pola, Sofía Paz-Sedano, Armando Macali, Dan Minchin, Agnese Marchini, Fabio Vitale, Cataldo Licchelli, Fabio Crocetta

**Affiliations:** 1 Departamento de Biología, Edificio de Biología, Universidad Autónoma de Madrid, Campus de Excelencia Internacional UAM + CSIC, Madrid, Spain; 2 Centro de Investigación en Biodiversidad y Cambio Global (CIBC-UAM), Campus de Excelencia Internacional UAM + CSIC, Madrid, Spain; 3 Dipartimento di Ecologia Marina Integrata (EMI), Stazione Zoologica Anton Dohrn, Napoli, Italy; 4 Department of Ecological and Biological Sciences, Ichthyogenic Experimental Marine Centre (CISMAR), Tuscia University, Tarquinia, Viterbo, Italy; 5 Marine Research Institute, Klaipėda University, Klaipėda, Lithuania; 6 Marine Organism Investigation, Marina Village, Ballina, Killaloe, County Clare, Ireland; 7 Department of Earth and Environmental Sciences, University of Pavia, Pavia, Italy; 8 Museo Civico di Storia Naturale del Salento, Calimera, Lecce, Italy; 9 Associazione Salento Sommerso, Lecce, Italy; 10 Hydracoop – Istituto di Ricerca per la Pesca e l'Acquacoltura, Lecce, Italy; University of California, UNITED STATES

## Abstract

The precise number of *Okenia* taxa inhabiting the Mediterranean Sea, as well as their general taxonomy, varies according to different specialists. So far, eight valid species have been reported from the area: *Okenia aspersa* (Alder & Hancock, 1845), *Okenia cupella* (Vogel & Schultz, 1970), *Okenia elegans* (Leuckart, 1828), *Okenia hispanica* Valdés & Ortea, 1995, *Okenia impexa* Er. Marcus, 1957, *Okenia leachii* (Alder & Hancock, 1854), *Okenia mediterranea* (Ihering, 1886), and *Okenia zoobotryon* (Smallwood, 1910). Of these, only three (*O*. *elegans*, *O*. *hispanica*, and *O*. *mediterranea*) have their type localities in the Mediterranean Sea, whereas the others were described from different biogeographic areas and later included in the Mediterranean biota. We carried out a review on Mediterranean *Okenia* species through an integrative approach, based on a wide literature search and a morphological and molecular analysis of available type material and samples collected recently. The present study confirmed the presence of *O*. *aspersa*, *O*. *elegans*, *O*. *hispanica*, and *O*. *mediterranea* in the Mediterranean Sea, although leaving remaining questions about some of those taxa. The distribution of *O*. *cupella*, *O*. *impexa*, and *O*. *zoobotryon* is limited to the western Atlantic, and of *O*. *leachii* to the eastern Atlantic. All specimens previously identified as *O*. *cupella*, *O*. *impexa*, and *O*. *zoobotryon* by different authors in the Mediterranean Sea were repeatedly misidentified. Thus, we describe *Okenia problematica* sp. nov. and *Okenia longiductis* sp. nov., from the “Mediterranean” *Okenia cupella/impexa* and *O*. *zoobotryon*. We also consider here *Okenia pusilla* Sordi, 1974 a *nomen dubium* and include a redescription of the holotype of *O*. *cupella*. A molecular phylogeny, including all the sequenced *Okenia* species, was performed in order to evaluate the evolutionary relationships of the newly described species with the other congeneric taxa.

## Introduction

The genus *Okenia* Menke, 1830 (Gastropoda: Nudibranchia: Goniodorididae) includes around 50 valid species worldwide and is composed by small to medium-sized sea slugs, whose distribution spans from cold, temperate, and tropical waters of the Pacific Ocean to the north and south Atlantic Ocean, including the Mediterranean Sea, and bathymetric range goes from the intertidal to the 160 meters depths of *Okenia vancouverensis* (O´Donoghue, 1921) [1–6]. Little is known about the phylogenetic relationships between *Okenia* and related genera. Gosliner [2] synonymized *Hopkinsia* MacFarland, 1905, *Hopkinsiella* Baba, 1938, and *Sakishimaia* Hamatani, 2001 with *Okenia* based on morphological characters, while Pola et al. [7] and Paz-Sedano et al. [8] confirmed the monophyly of the genus *Okenia* and the synonymy of *Hopkinsia* and *Hopkinsiella* proposed by Gosliner [2] based on preliminary molecular data. An even more intricate situation concerns *Okenia* alpha taxonomy, since many new species of *Okenia* have been recently described [2–5, 7–13], some of these lack complete morphological descriptions [5, 12–13], and many new species still require description [14]. The validity of species identification has varied historically according to the views of different authors [2, 15–19], with cryptic or pseudocryptic species still being discovered at the beginning of the twenty-first century [7]. This makes the known distribution of previously described *Okenia* species uncertain, thereby creating confusion of true geographic ranges [5, 13, 18]. As a result, the overall knowledge of *Okenia* is incomplete, and reviews focusing on selected biogeographic areas and determining the true identity and spread of *Okenia* taxa worldwide need to be undertaken through both genetic and anatomical studies.

The precise number of *Okenia* species inhabiting the Mediterranean Sea has also varied according to different specialists. While the Mediterranean malacofauna is generally considered to be one of the best studied worldwide [20–21], the origin of the modern Mediterranean molluscan assemblage is complex [20–23], which often lead to the discovery of cryptic diversity even in well-known groups [24–29]. This is compounded further by potentially similar species introduced by anthropogenic activities from the Atlantic and Indo-Pacific bioregions [30–31].

Here we carry out a review on the Mediterranean *Okenia* species using an integrative approach, based on the information existing in the literature, supplemented by morphological and molecular analysis of samples newly collected and type material from the Mediterranean Sea and outside. A molecular phylogeny including sequenced *Okenia* species was also undertaken to elucidate the evolutionary relationships of the newly collected specimens with the other congeneric taxa.

## Material and methods

### Published data and source of newly collected specimens

Indexed and grey literature were examined for published Mediterranean records of taxa belonging to the genus *Okenia*, especially those accounts concerning faunistic, taxonomic, and biogeographic studies of Mollusca. Bibliographic data were critically analysed and taxonomically updated to the latest nomenclature available, following the World Register of Marine Species [32]. At the same time, a GenBank search was carried out to check for barcodes of *Okenia* material from the Mediterranean Sea. Once these two preliminary steps were achieved, *Okenia* specimens were collected from marinas by hand or from several Mediterranean localities by SCUBA diving, and preserved in 96–100% ethanol. All examined material was deposited either at the Museo Nacional de Ciencias Naturales (MNCN, Madrid, Spain) or Stazione Zoologica Anton Dohrn (SZN, Naples, Italy). In addition, we borrowed two specimens of *Okenia angelensis* Lance, 1966 and the holotype of *Okenia cupella* (Vogel & Schultz, 1970) from the California Academy of Science (CASIZ, San Francisco, California) and the Smithsonian National Museum of Natural History (USNM, Washington D. C., United States) respectively, in order to add them to our dataset and thus allow for morphological and molecular comparisons with our collections.

### Morphological examination

The external morphology of specimens was examined from photographs of living *Okenia* specimens and from laboratory observations. Internal organs were removed following a dorsal incision and drawn using a Nikon SMZ-1500 dissecting microscope with an attached camera lucida. Special attention was paid to the buccal mass and the reproductive system. Each buccal mass was removed and dissolved in 10% sodium hydroxide to remove surrounding tissue. The labial cuticle and radula were then rinsed in water. These structures and the penis were initially examined under the light microscope, then photographed using the software *cellSense*, and subsequently dried (apart from the radula) by critical point using hexamethyldisilazane. All these parts were finally mounted and sputter coated for examination under a Hitachi S3000N scanning electron microscope (SEM).

### DNA extraction, amplification and sequencing

DNA was extracted from foot tissue and performed using the DNeasy Blood and Tissue Kit (Qiagen) according to the manufacturer’s instructions or by proteinase K-digestion followed by a standard phenol-chloroform protocol [33]. Partial sequences of cytochrome c oxidase I (COI), 16S ribosomal RNA (16S rRNA), and histone H3 (H3) were amplified using LCO1490 and HCO2198 universal primers for COI [34], 16S ar-L and 16S br-H for 16S rRNA [35], and H3AD5’3’ and H3BD5’3’ for H3 [36]. For the DNA extracted using the DNeasy Blood and Tissue Kit, the master mix for the PCR was prepared in the following order: nuclease-free water up to 25 μl volume reaction, 2.5 μl of Qiagen buffer, 2.5 μl of dNTP (2 mM), 5 μl of ‘Q-solution’ (Qiagen), 1.5–3.5 μl magnesium chloride (25 mM), 0.5 μl of each forward and reverse primer (10 mM), 1 μl of DNA polymerase (250 units), and 2.5 μl of DNA. Amplifications were performed with an initial denaturation for 5 min at 95 °C, followed by 35 cycles of 30 s at 95 °C, 30–45 s annealing at 49 °C for COI, 52 °C for 16S rRNA, and 50 °C for H3, and 45 s at 72 °C with a final extension of 5 min at 72 °C. For the DNA extracted using the phenol-chloroform protocol, the master mix for the PCR was prepared in the following order: nuclease-free water up to 15 μl volume reaction, 3 μl of Promega buffer, 0.3 μl of dNTP (10 mM), 1 μl magnesium chloride (25 mM), 0.3 μl of each forward and reverse primer (10 mM), 0.1 μl of Promega HotStart DNA polymerase (2 units), and 1.5 μl of DNA. Amplifications were performed for COI with an initial denaturation for 2 min at 94 °C, followed by 35 cycles of 60 s at 94 °C, 60 s annealing at 48 °C and 90 s at 72 °C, with a final extension of 10 min at 72 °C. For 16S rRNA amplifications were performed with an initial denaturation for 2 min at 94 °C, followed by 35 cycles of 45 s at 94 °C, 60 s annealing at 51 °C and 90 s at 72 °C, with a final extension of 10 min at 72 °C. Finally, H3 amplifications were performed with an initial denaturation for 2 min at 94 °C, followed by 40 cycles of 30 s at 94 °C, 30 s annealing at 54 °C and 60 s at 72 °C, with a final extension of 10 min at 72 °C. Successful PCR products were purified and sequenced by Macrogen, Inc.

### Phylogenetic analyses

Sequences were assembled and edited using Bioedit v7.2.5 [37] and aligned using MEGA6 [38]. Protein-coding sequences were translated into amino acids for confirmation of alignment using the genetic code invertebrate mitochondrial DNA for COI and universal code for H3. All sequences were blasted in GenBank to check for contamination. The most variable regions from the 16S rRNA alignment were removed by using both the default settings and the standard options for stringent and less stringent selection in Gblocks [39]. Excluding “indel-rich” regions, the tree was in general the same. Therefore, final analyses were performed with all bases included. Sequences of COI, 16S rRNA, and H3 were trimmed to 658, 470, and 328 base pairs, respectively. The evolutionary models were selected using jModelTest-2.1.7 [40] under the Bayesian information criteria [41]. For COI and H3 the evolutionary model was determined separately for the first, second, and third codon position. Evolutionary models for COI were TIM2+I+G, TPM3uf+I, and TrN+I+G for the first, second, and third codon position, respectively. The TPM1uf+I+G evolutionary model was selected for 16S rRNA gene. For H3 gene, TIM2+I was selected for first codon position, JC for second codon position, and TVM+G for third codon position. Bayesian Inference (BI) and Maximum Likelihood (ML) analyses were conducted for individual genes as well as for the concatenate of a minimum of two (COI+16S rRNA) (H3+COI) to three genes (H3+COI+16S rRNA). BI analysis was performed using the software package MrBayes v3.1.2b [42] for ten million generations with two independent runs and sampling frequency of 1000. ML analysis was performed using the software package RAxML v7.04 [43]. To determine the nodal support in ML a 50000 bootstrap analysis was implemented. Only nodes supported by bootstraps values ≥ 75 [44] and posterior probabilities ≥ 0.96 were considered statistically significant [45]. The trees obtained were visualized in FigTree v1.3.1 [46] and edited in Adobe Photoshop CC 2014.

### Species delimitation analyses

In order to compare the genetic distances amongst specimens of *Okenia* included in this study, we calculated the pairwise uncorrected *p*-distances for COI and H3 using PAUP*4.0b 10.0 [47]. All codon positions were considered for the analysis. Analyses of species delimitation—Bayesian Poisson Tree Process (bPTP) [48] and Automatic Barcode Gap Discovery (ABGD) [49]—were conducted on the COI ingroup sequences. bPTP analysis was done using the bPTP webtool (https://species.h-its.org), running 200000 MCMC generations, Thinning = 100 and Burn-in = 0.1. ABGD analysis was run using Kimura (K80) evolutive model, a relative gap width (X) = 1, a divergence of intraspecific diversity between 0.0001 and 0.1 and Nb bins = 20. The matrix was loaded into the online ABGD webtool (http://wwwabi.snv.jussieu.fr/public/abgd/abgdweb.html).

### Nomenclatural acts

The electronic edition of this article conforms to the requirements of the amended International Code of Zoological Nomenclature, and hence the new names contained herein are available under that Code from the electronic edition of this article. This published work and the nomenclatural acts it contains have been registered in ZooBank, the online registration system for the ICZN. The ZooBank LSIDs (Life Science Identifiers) can be resolved and the associated information viewed through any standard web browser by appending the LSID to the prefix “http://zoobank.org/”. The LSID for this publication is urn:lsid:zoobank.org:pub:5F42073C-02B6-458E-99CE-F4814D959E3C. The electronic edition of this work was published in a journal with an ISSN, and has been archived and is available from the following digital repositories: PubMed Central and LOCKSS.

## Results

### Published data of *Okenia* taxa living in the Mediterranean Sea to date

**Fig 1:**
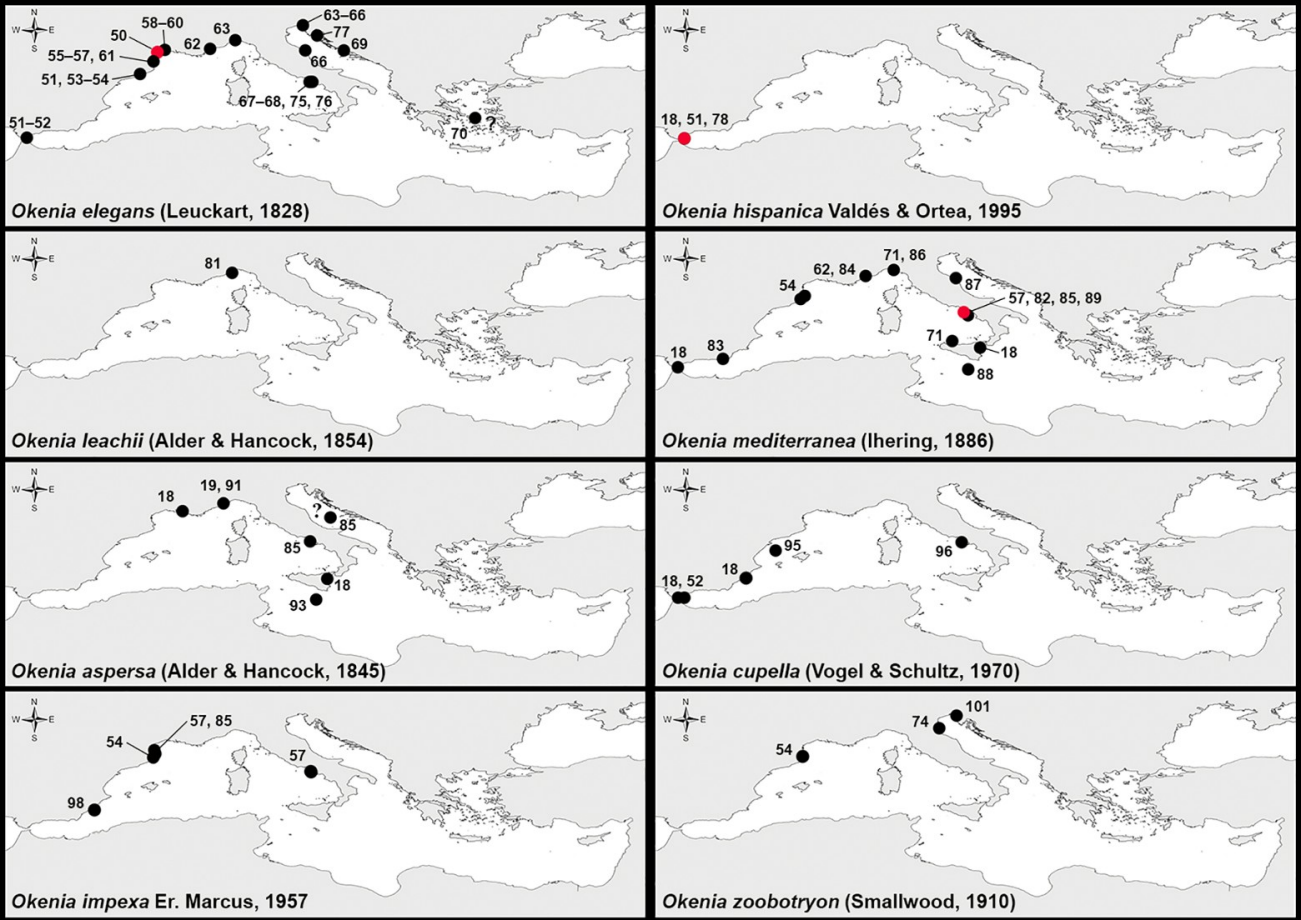
*Okenia* taxa recorded from the Mediterranean Sea. Locality numbers as in Table 1. Type localities of species described from the Mediterranean Sea marked with a red dot. When the exact locality was unknown, a dot was placed in the middle of the sighting area accompanied by a question mark.

The literature analysis revealed records of several valid taxa, some of which were considered to be misidentifications, and some belonging to taxa now considered as junior synonyms (Table 1; Fig 1). To date, the *Okenia* taxa considered as valid and recorded from the Mediterranean Sea can be divided into two groups according to their external colour patterns. This is based on those with striking colours (4 taxa), and those with whitish/brownish tonalities (4 taxa) (Table 1).

**Table 1:** Literature records of *Okenia* taxa known from the Mediterranean Sea (valid taxa marked in bold), with type locality or listed localities (TL), Mediterranean records based on concrete material, presence in recent (1990–now) review/books listing Mediterranean biota as a whole (R/B), and notes (N). Numbers as in Fig 1.

Nominal taxon	TL	Records	R/B	N
**SPECIES WITH STRIKING COLOUR PATTERN**				
***Okenia elegans* (Leuckart, 1828)**	Cette, France (Mediterranean Sea) [50]	**Spain**: Estrecho de Gibraltar [51–52]; Cataluña area [51, 53–54]. **France**: Cette [50]; Banyuls-sur-mer [55–57]; Marseille [58–60]; Port-Vendres [61]; Villefranche-sur-mer [62]. **Italy**: Trieste [63–66]; Genova [63]; Naples area [67–68]; Conero area [66]. **Croatia**: Split [69]. **Greece**: North Aegean [70]	[23, 71–74]	
= *Idalia dautzenbergi* Vayssière, 1919	Marseille, France (Mediterranean Sea) [59]	**France**: Marseille [59]		
= *Euplocamus cirriger* Philippi, 1839	Naples, Italy (Mediterranean Sea) [75]	**Italy**: Naples [75]		
= *Euplocamus laciniosus* Philippi, 1841	Naples, Italy (Mediterranean Sea) [76]	**Italy**: Naples [76]. **Croatia**: Mali Lošinj [77]		
***Okenia hispanica* Valdés & Ortea, 1995**	Mar de Alborán, Spain (Strait of Gibraltar) [18, 51, 78]	**Spain**: Mar de Alborán [18, 51, 78]	[23, 73–74]	
***Okenia leachii* (Alder & Hancock, 1854)**	Torbay, Great Britain (Atlantic Ocean) [79–80]	**Italy**: Mar Ligure occidentale [81]	[71–74]	
***Okenia mediterranea* (Ihering, 1886)**	Naples, Italy (Mediterranean Sea) [82]	**Spain**: Tarifa [18]; Almeria [83]; Illes Medes [54]; Platja d’Aro [54]. **France**: Villefranche-sur-mer [62, 84]. **Italy**: Naples area [57, 82, 85]; Portofino [71, 86]; Capo Gallo, Palermo [71]; Aci Trezza [18]; Conero area [87]. **Malta**: off Ghajn [88]	[23, 71–74]	
= *Okenia amoenula* Bergh, 1907 *sensu* [89]	Gordon’s Bay, Republic of South Africa (Atlantic Ocean) [90]	**Italy**: Naples area [89]		1
**SPECIES WITH WHITISH/BROWNISH TONALITIES**				
***Okenia aspersa* (Alder & Hancock, 1845)**	Cullercoats, Great Britain (North Sea) [15]	**Italy**: Noli [19, 91]	[74]	
= *Doris quadricornis* Montagu, 1815	South Devonshire, Great Britain (English Channel) [92]	**France**: Marseille *fide* [18]. **Italy**: Ischia [85]; Mar Adriatico [85]; Aci Trezza [18]. **Malta**: Golden Bay [93]	[71–72]	2
***Okenia cupella* (Vogel & Schultz, 1970)**	York River, Virginia (Atlantic Ocean) [94]	**Spain**: Cabo de Palos, Murcia [18]; Islas Columbretes [95]; Estrecho de Gibraltar [18, 52]	[23, 74]	3
= *Okenia pusilla* Sordi, 1974	Ischia, Italy (Mediterranean Sea) [96]	**Italy**: Ischia [96]		4
***Okenia impexa* Er. Marcus, 1957**	São Sebastião, Brasil (Atlantic Ocean) [97]	**Spain**: Cabo de Palos, Murcia [98]; L’Escala, Palamós [54]. **France**: Banyuls-sur-mer [57, 85]. **Italy**: Naples [57]	[71–73]	3
= *Okenia impexa banyulensis* Schmekel, 1979 (not available under ICZN rules, see notes)	Banyuls-sur-mer (France) and Naples (Italy) (Mediterranean Sea) [57]	**France**: Banyuls-sur-mer [57]. **Italy**: Naples [57]		5
***Okenia zoobotryon* (Smallwood, 1910)**	Hamilton Parish, Bermuda (Great Britain) (Atlantic Ocean) [99–100]	**Spain**: Cala Maset, Sant Feliu de Guíxols [54]. **Italy**: Pialassa Baiona, Ravenna [74]. **Slovenia**: entire coastline [101]	[74]	

Notes:

^1)^ The only record of *Okenia amoenula* Bergh, 1907 from the Mediterranean Sea is based on [89]. However, the same author subsequently corrected herself and considered it as a misidentification for *Okenia mediterranea* (Ihering, 1886) [57].

^2)^ *Doris quadricornis* Montagu, 1815 was suppressed under the plenary powers for the purpose of the Principle of Priority in 1974 under ICZN Opinion 1014 [102]. The valid name for this species is *Okenia aspersa* (Alder & Hancock, 1845).

^3)^ *Okenia cupella* (Vogel & Schultz, 1970) and *Okenia impexa* Er. Marcus, 1957 have a troublesome taxonomic and biogeographic history. In fact, despite being different valid taxa [103–104], they were sometimes considered synonym [105]. In addition, Mediterranean records were assigned to one or the other taxon according to different authors’ points of views, despite dealing with a single entity [5, 18, 51–52, 57, 74]. Such a debate seemed to be originally solved by [18], with several subsequent authors considering Mediterranean records of *O*. *impexa* to be ascribed to *O*. *cupella* [5, 23, 51–52], until [73] listed *O*. *impexa* as part of the Mediterranean biota and excluded *O*. *cupella* with no rationale, and [53] recorded *Okenia* cf. *impexa* from the Mediterranean coast of Spain, again with no explainations or discussions on differences from *O*. *cupella*. Owing to this confusion, we kept Mediterranean literature records separated according to the binomial name used in the original source.

^4)^ *Okenia pusilla* Sordi, 1974 was originally considered a synonym of *Okenia impexa* Er. Marcus, 1957 by [85], until [18] considered it a synonym of *Okenia cupella* (Vogel & Schultz, 1970) [103–104], and therefore we kept it according to the latest published statements. However, see also note 3 for further taxonomic problems on *O*. *cupella* and *O*. *impexa*.

^5)^ This putative subspecies was only conditionally proposed by [57]. However, according to ICZN [106] rules (art. 15.1), a new name or nomenclatural act proposed conditionally and published after 1960 is not thereby made available.

The group with striking colours is the “easiest” to determine and consists of three taxa originally described from the Mediterranean Sea: *Okenia elegans* (Leuckart, 1828) (type species of *Okenia* Menke, 1830), *Okenia hispanica* Valdés & Ortea, 1995, and *Okenia mediterranea* (Ihering, 1886), and three junior synonyms of *O*. *elegans*, namely *Idalia dautzenbergi* Vayssière, 1919, *Euplocamus cirriger* Philippi, 1839, and *Euplocamus laciniosus* Philippi, 1841, described from the Mediterranean Sea (Table 1; Fig 1). The other species of this group, *Okenia leachii* (Alder & Hancock, 1854), was originally described from Torbay, Great Britain (Atlantic Ocean). Our literature research revealed that the presence of *O*. *leachii* in the Mediterranean Sea is doubtful and should be rejected. It was first listed as occurring in the Mediterranean Sea by Ihering [82], but such a statement was then either ignored [63, 107] or questioned [58] by subsequent authors and was finally dismissed since it was not based on preserved material. Then, a single specimen from the western Ligurian Sea was collected and published as an alleged “new record from the Mediterranean Sea” by Cattaneo-Vietti [81] (Table 1; Fig 1), but this was not supported by either a photograph or preserved material, and is now considered doubtful by Cattaneo-Vietti (pers. comm.) himself. The only photograph of *O*. *leachii* published in the Mediterranean literature, or in Mediterranean Sea slug websites, is in Trainito & Doneddu [74], which shows a specimen from Eilean Siar (Great Britain, Atlantic Ocean) (B. Picton, pers. comm.). This exclusion reduces the number of valid *Okenia* species with striking colours recorded from the Mediterranean to three.

The group with external colouration of white or brownish tonality, with the exception of *Okenia aspersa* (Alder & Hancock, 1845), has had a troublesome taxonomic and biogeographic history. Two species, *Okenia cupella* (Vogel & Schultz, 1970) and *Okenia impexa* Er. Marcus, 1957, were recorded for the Mediterranean basin but not originally described from the area. As explained in Note 3 of Table 1, these two species are valid at their original type localities. However, the Mediterranean records are contradictory and records ascribed to those two taxa in the Mediterranean simply belong to a single entity (Table 1 and notes therein). Two separate taxa (*Okenia pusilla* Sordi, 1974 and *Okenia impexa banyulensis* Schmekel, 1979), both described from the Mediterranean Sea, are now considered a junior synonym and an invalid introduction, respectively (Table 1 and notes 4, 5). Finally, *Okenia zoobotryon* (Smallwood, 1910), a species associated with the supposedly worldwide distributed bryozoan *Amathia verticillata* (delle Chiaje, 1822), was also recently recorded as a non-indigenous species within the Mediterranean Sea based on external morphology only [54, 74, 101] (Table 1; Fig 1). However, more or less simultaneously, Pola [100] reviewed the worldwide taxonomy and distribution of *Okenia zoobotryon*, showing that it is confined to its type locality region, ranging from Bermuda to Cuba (Western Atlantic Ocean). Indeed, these latter Mediterranean records could be explained by human-mediated introductions; however, further examination of Mediterranean specimens is required, since external morphology may be highly misleading in this taxon [100].

### Recentely collected *Okenia* taxa living in the Mediterranean Sea and data mining from GenBank

Our field collections provided four different taxa. Two of them were clearly identified as *Okenia elegans* (Leuckart, 1828) and *Okenia mediterranea* (Ihering, 1886). However, despite their external similarities, the remaining taxa could not be clearly ascribed either to *Okenia zoobotryon* or *Okenia cupella*/*impexa*. For this reason, we listed our specimens as *Okenia* sp. 1 and *Okenia* sp. 2 until the molecular and morphological studies were undertaken below (Table 2). Those specimens morphologically examined are listed in the systematic section, whilst our recently sequenced specimens are listed in Table 3.

**Table 2:** *Okenia* specimens from the Mediterranean Sea recently collected and used in the present study. Species, number of specimens (N), vouchers, locality and coordinates, collector, and collection date.

Preliminary identification	N	Vouchers	Locality	Coordinates	Collector	Date
*Okenia elegans*	1	MNCN15.05/88175	La Planassa (Girona, Spain)	41°35'07.60"N—02°57'59.30"E	Manuel Ballesteros	28.04.2016
*Okenia mediterranea*	1	MNCN15.05/88174	Santa Maria al Bagno (Lecce, Italy)	40°07'13.39"N—17°59'56.75"E	Fabio Vitale	28.05.2016
*Okenia* sp. 1	8	MNCN15.05/20037 MNCN15.05/88166–68 SZN-MOL0005 SZN-MOL0012–14	Mar Piccolo (Taranto, Italy)	40°28'26.00"N—17°16'18.00"E	Enrico Ricchitelli	29.11.2015
*Okenia* sp. 1	9	MNCN15.05/200040–42 MNCN15.05/888171 SZN-MOL0008–9 SZN-MOL0017–19	Naples (Italy)	40°49'39.50"N—14°14'59.40"E	Armando Macali	07.12.2016
*Okenia* sp. 1	15	MNCN15.05/70411 MNCN15.05/200035–36 MNCN15.05/88164–65 SZN-MOL0003–4 SZN-MOL0010–11	Lago di Sabaudia (Latina, Italy)	41°15'03.70"N—13°02'30.20"E	Armando Macali	04.10.2015
*Okenia* sp. 1	7	MNCN15.05/200038–39 MNCN15.05/88169–70 SZN-MOL0006–7 SZN-MOL0015	Porto Ercole (Grosseto, Italy)	42°23'30.20"N—11°12'39.50"E	Armando Macali	11.11.2016
*Okenia* sp. 1	3	MNCN15.05/88172–73 SZN-MOL0016	La Grande-Motte (Hérault, France)	43°33'21.11"N—04°04'58.17"E	Dan Minchin & Agnese Marchini	19.11.2014
*Okenia* sp. 2	2	MNCN15.05/200034 SZN-MOL0001	Gallipoli (Lecce, Italy)	40°06'25.39"N—17°58'03.38"E	Fabio Vitale & Cataldo Licchelli	12.11.2015
*Okenia* sp. 2	2	MNCN15.05/88162 SZN-MOL0002	Aiguafreda (Barcelona, Spain)	41°57'50.00"N—03°13'42.06"E	Manuel Ballesteros	01.06.2010
*Okenia* sp. 2	1	MNCN15.05/88163	Cala Joncols (Girona, Spain)	42°15'04.10"N—03°15'35.30"E	Marina Poddubetskaia	09.07.2018

**Table 3:** Species (authorities not included) used for molecular analyses, voucher, locality, and GenBank accession numbers. Specimens sequenced during the present study marked with an asterisk.

Specie	Voucher	Locality	COI	16S	H3
*Berthella martensi*	MZUCR6982	Panama, Las Secas	HM162683	HM162592	HM162498
*Armina scotti*	CASIZ177534	Philippines, Batangas, Anilao, Luzon, Mainit Point	HM162696	HM162606	HM162512
*Leminda millecra*	CASIZ176348	South Africa, Percy’s Hole, Gordon’s Bay, Eastern False Bay	HM162745	HM162669	HM162578
*Bornella valdae*	CASIZ176832	South Africa, Natal Province, Durban	HM162706	HM162626	HM162532
*Sakuraeolis enosimensis*	CASIZ178876	California, Richardson Bay	HM162758	HM162682	HM162591
*Felimida edmundsi*	CASIZ179385	Gulf of Guinea, Sao Tome & Principe, Ilha do Principe	HM162686	HM162595	HM162501
*Felimida luteopunctata*	MNCN15.05/70688	Spain, Cádiz, Santa María Beach	KJ911282	KJ911262	KJ911241
*Triopha maculata*	CASIZ181556	California, Marin County, Duxbury Reef	HM162691	HM162601	HM162507
*Tyrannodoris ricei*	CASIZ173900	Florida, shore of Loran Tower	HM162688	HM162598	HM162504
*Tambja marbellensis*	CASIZ180379	Portugal, Setubal District, Outao	HM162689	HM162599	HM162505
*Armodoris anudeorum*	LACM3118	Antarctica, Ross Sea, McMurdo Sound	KP340387	KP340290	KP340412
*Diaphorodoris lirulatocauda*	CASIZ184341	California, Duxbury Reef, Marin Co	KP340403	KP340307	KP340422
*Diaphorodoris luteocincta*	LACM8.7A	Spain, Cádiz, Bahía de Algeciras	KP340404	KP340308	KP340423
*Corambe obscura*	CASIZ183942	New Hampshire, Rockingha, New Castle Portsmouth Bay	KP340399	KP340303	KP340419
*Corambe pacifica*	LACM2007-2.6B	California, Los Angeles, Long Beach Marina	KP340400	KP340304	KP340420
*Knoutsodonta jannae*	CASIZ175578	California, San Mateo, Pillar Point	KP340392	KP340296	KP340415
*Onchidoris proxima*	CASIZ183921A	Maine, Washington, Passamaquody Bay Eastport	KM219676	KJ653673	KM225826
*Trapania hispalensis*	MZCN15.05/55504	Portugal, Aveiro	JX274080	JX274048	-
*Trapania reticulata*	CASIZ191431	Papua New Guinea, Tab Island	MF958432	MF958303	-
*Ancula gibbosa*	CASIZ182028	Maine, Cumberland	KP340388	KP340291	KP340413
*Goniodoris castanea*	-	Sweden, Bohuslan, Kristineberg	AJ223263	AJ225187	-
*Goniodoris nodosa*	-	Spain, NE Atlantic	AF249788	AF249226	-
*Okenia amoenula*	CASIZ176191	South Africa, Western Cape Province, False Bay, Gordons Bay	KF192606	-	KF744248
*Okenia aspersa*	MNCN15.05/70410	France, Cape Ferret	KY661374	KY661368	KY661382
*Okenia brunneomaculata*	CASIZ177712	Philippines, Luzon Island, Balayan Bay	KF744236	-	KF744242
*Okenia felis*	CASIZ174175	California, Monterey County, Point Lobos	KF744237	-	KF744243
*Okenia harastii*	MNCN15.05/46986	Australia, Nelson Bay, Port Stephen	KF744238	-	KF744244
*Okenia pellucida*	MNCN15.05/46987	Australia, Nelson Bay, Port Stephens	KF744239	-	KF744245
*Okenia picoensis*	MNCN15.05/60181	Azores, Pico Island	KY661376	KY661370	-
*Okenia rosacea*	CASIZ184340	California, Marin County, Duxbury Reef	KF192605	-	KF744249
*Okenia vena*	MNCN15.05/70408	Australia, Nelson Bay	KY661380	KY661372	KY661383
*Okenia vena*	MNCN15.05/70409	Australia, Nelson Bay	KY661381	KY661373	KY661384
*Okenia zoobotryon*	CASIZ181105	Bermudas, Hamilton Parish, Tom Moore Pond	KF744241	-	KF744247
*Okenia angelensis	CASIZ208935	California, San Francisco	MK645757	MK650419	MK659664
*Okenia angelensis	CASIZ218828	California, San Francisco	MK645758	MK650420	MK659665
*Okenia cupella	USNM679396	Virginia, York River	-	-	MK659666
*Okenia elegans	MNCN15.05/88175	Spain, La Planassa	MK645759	MK650421	MK659667
*Okenia mediterranea	MNCN15.05/88174	Italy, Santa Maria al Bagno	MK645760	MK650422	MK659668
*Okenia sp. A	MNCN15.05/70411	Italy, Lago di Sabaudia	KY661379	KY661371	MK659669
*Okenia sp. 1	SZN-MOL0003	Italy, Lago di Sabaudia	MK645761	MK650423	MK659670
*Okenia sp. 1	SZN-MOL0004	Italy, Lago di Sabaudia	MK645762	MK650424	MK659671
*Okenia sp. 1	MNCN15.05/200035	Italy, Lago di Sabaudia	MK645763	MK650425	MK659672
*Okenia sp. 1	MNCN15.05/200036	Italy, Lago di Sabaudia	MK645764	MK650426	MK659673
*Okenia sp. 1	SZN-MOL0005	Italy, Mar Piccolo	MK645765	MK650427	MK659674
*Okenia sp. 1	MNCN15.05/200037	Italy, Mar Piccolo	MK645766	-	-
*Okenia sp. 1	SZN-MOL0013	Italy, Mar Piccolo	MK645767	MK650428	-
*Okenia sp. 1	SZN-MOL0006	Italy, Porto Ercole	MK645768	MK650429	MK659675
*Okenia sp. 1	SZN-MOL0015	Italy, Porto Ercole	MK645769	-	MK659676
*Okenia sp. 1	MNCN15.05/88169	Italy, Porto Ercole	MK645770	-	MK659677
*Okenia sp. 1	MNCN15.05/88170	Italy, Porto Ercole	MK645771	-	MK659678
*Okenia sp. 1	MNCN15.05/200038	Italy, Porto Ercole	MK645772	MK650430	MK659679
*Okenia sp. 1	SZN-MOL0007	Italy, Porto Ercole	MK645773	MK650431	MK659680
*Okenia sp. 1	MNCN15.05/200039	Italy, Porto Ercole	MK645774	MK650432	MK659681
*Okenia sp. 1	MNCN15.05/200040	Italy, Naples	MK645775	MK650433	MK659682
*Okenia sp. 1	SZN-MOL0008	Italy, Naples	MK645776	MK650434	MK659683
*Okenia sp. 1	SZN-MOL0019	Italy, Naples	MK645777	-	MK659684
*Okenia sp. 1	MNCN15.05/200041	Italy, Naples	MK645778	MK650435	MK659685
*Okenia sp. 1	SZN-MOL0017	Italy, Naples	MK645779	-	MK659686
*Okenia sp. 1	SZN-MOL0009	Italy, Naples	MK645780	MK650436	MK659687
*Okenia sp. 1	SZN-MOL0018	Italy, Naples	MK645781	-	MK659688
*Okenia sp. 1	MNCN15.05/88171	Italy, Naples	MK645782	-	MK659689
*Okenia sp. 1	MNCN15.05/200042	Italy, Naples	MK645783	MK650437	MK659690
*Okenia sp. 2	MNCN15.05/200034	Italy, Gallipoli	MK645784	-	MK659691
*Okenia sp. 2	SZN-MOL0001	Italy, Gallipoli	MK645785	-	MK659692

GenBank revealed sequences available for three taxa recorded from the Mediterranean Sea: *Okenia aspersa* (Alder & Hancock, 1845) (COI, 16S, and H3), *Okenia zoobotryon* (Smallwood, 1910) (COI and H3), and *Okenia* sp. A (COI and 16S). *Okenia aspersa* was originally described from Cullercoats (England) in the North Sea [16], and the sequenced specimen come from Cape Ferret on the Atlantic coast of France, while *Okenia zoobotryon* was originally described from Bermuda [99–100], and the sequences come from its type locality. The specimen identified as *Okenia* sp. A was collected from Sabaudia Lake (Italy) and already partially sequenced in Paz-Sedano et al. [8]. In this study, we added the H3 sequence and revealed its true identity. Moreover, several additional sequences of species belonging to the genus *Okenia* and the family Goniodorididae were retrieved from GenBank to compare our results and the evolutionary relationships of the newly barcoded taxa, as well as species to be used as outgroups (see Table 3).

### Molecular results

**Fig 2:**
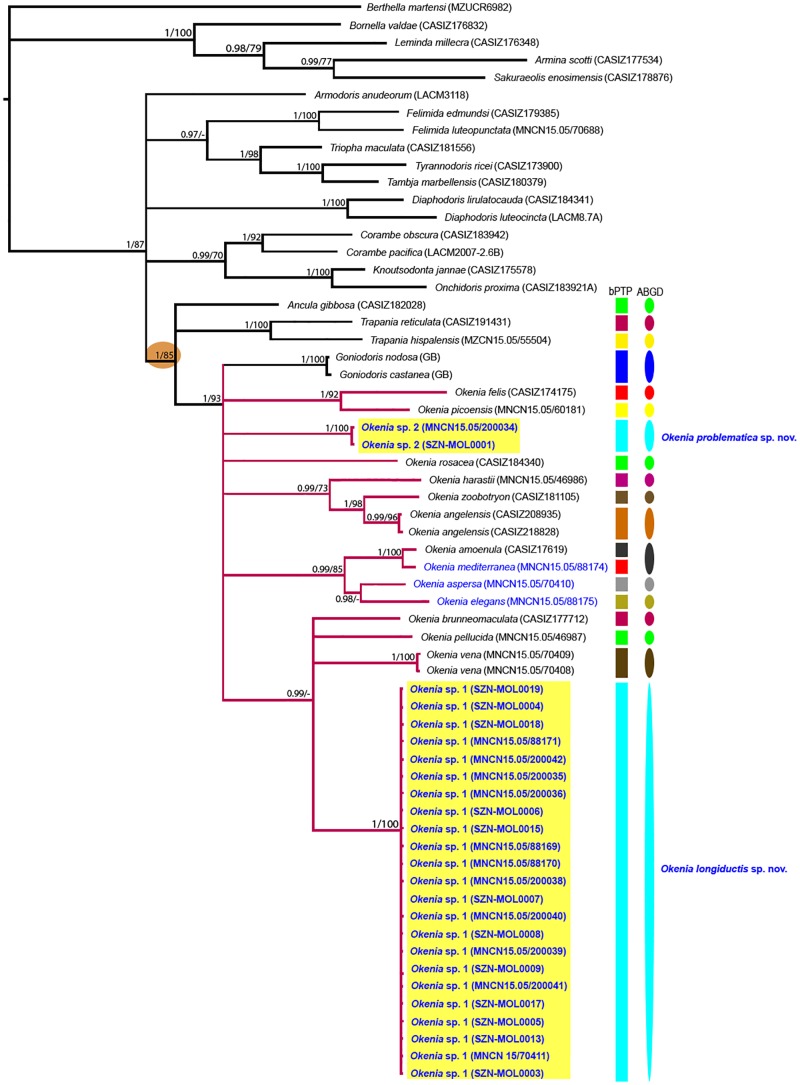
Phylogenetic relationships (BI, ML) based on the concatenated mitochondrial (COI and 16S rRNA) and nuclear (H3) genes. Orange circle indicates Goniodorididae taxa. Pink branches represent *Okenia* taxa. *Okenia* species living in the Mediterranean Sea highlighted in blue. New species identified in the present study highlighted in yellow. Different colours highlighted in bPTP and ABGD species delimitation analyses represent potential different taxa.

The phylogenetic tree based on concatenated genes sequences including all species of *Okenia* Menke, 1830 and other taxa belonging to Goniodorididae H. Adams & A. Adams, 1854 is shown in Fig 2. All taxa belonging to Goniodorididae (highlighted with an orange circle) are clustered in a well-supported clade (BI = 1, ML = 85) (Fig 2). The resulted tree showed a polytomy including the two *Goniodoris* species included in the analysis and *Okenia* species, suggesting that their phylogenetic relationship is still unresolved (BI = 1, ML = 93) (Fig 2).

With respect to the species of the genus *Okenia*, our analyses have not been able to clarify all the existing relationships among the species included. However, we identified four main clades: *i*) the first one clusters *Okenia felis* Gosliner, 2010 and *Okenia picoensis* Paz-Sedano, Ortigosa & Pola, 2017 (BI = 1, ML = 92) (Fig 2); *ii*) the second includes *Okenia zoobotryon* (Smallwood, 1910), *Okenia harastii* Pola, Roldán & Padilla, 2014, and *Okenia angelensis* Lance, 1966 (BI = 0.99, ML = 73), with *O*. *zoobotryon* and *O*. *angelensis* as sister species (BI = 1, ML = 98) (Fig 2); *iii*) the third clade includes *Okenia amoenula*, *Okenia mediterranea*, *Okenia aspersa*, and *Okenia elegans* (BI = 0.99, ML = 85), with *O*. *amoenula* being sister species of *O*. *mediterranea* (Fig 2), and *O*. *aspersa* sister species of *O*. *elegans* (BI = 0.98) (Fig 2); *iv*) the last supported clade includes *Okenia brunneomaculata* Gosliner, 2004, *Okenia pellucida* Burn, 1967, *Okenia vena* Rudman, 2004, and *Okenia* sp. 1, with a BI value of 0.99 (Fig 2).

*Okenia* sp. 1 from Lago di Sabaudia, Mar Piccolo, Porto Ercole, and Naples (Mediterranean Sea) and *Okenia* sp. 2 from Gallipoli (Mediterranean Sea) are respectively grouped in single clades (Fig 2). Among the *Okenia* taxa retrieved from Genbank, *Okenia* sp. A (MNCN 15/70411) deserves specific mention. That specimen, originating from Lago di Sabaudia (Italy) and previously barcoded in a paper including some of us as co-authors (SPS and MP) [8], proved to be conspecific with *Okenia* sp. 1 (Fig 2). Our bPTP and ABGD species delimitation analyses clearly support that *Okenia* sp. 1 and *Okenia* sp. 2 belong to single entities each (Fig 2), with a COI uncorrected *p*-distance between them of 17.4–17.9% (Table 4). Interestingly, despite their external similarities, *Okenia* sp. 1 did not prove to be conspecific with either the topotypical *O*. *zoobotryon* or its sister species *Okenia angelensis* Lance, 1966. In addition, despite the fact that no material of *Okenia impexa* was available to us (see below for further discussion on this taxon) and the COI sequence of *Okenia cupella* was not obtained, the H3 analysis showed that *Okenia* sp. 2 is not conspecific with the holotype of *Okenia cupella*, with an uncorrected *p*-distance between these two taxa of 11.6% (Table 4). Indeed, it should be noted that all the Mediterranean taxa analysed here, except *Okenia* sp. 1 and *Okenia* sp. 2, were represented by single specimens due to their rarity; this somehow makes the results from the species delimitation analysis (ABGD and bPTP) less powerful then usual. However, on the other hand, both morphological comparisons and molecular results point toward the fact that *Okenia* sp. 1 and *Okenia* sp. 2 are undescribed species; thus, they are formally described below in the Systematics section. At the same time, as to allow for comparisons, also the holotype of *O*. *cupella* is redescribed here.

**Table 4:** COI and H3 gene pairwise uncorrected *p*-distances (%) amongst different species of *Okenia* and *Goniodoris*.

Species	COI *p*-distances (%)	H3 *p-*distances (%)
*Goniodori*s *nodosa vs Goniodoris castanea*	0.7	-
*Okenia* sp. A *vs Okenia* sp. 1	0.3	0
Between specimens of *Okenia* sp. 1	0–0.7	0
Between specimens of *Okenia* sp. 2	1	0
*Okenia* sp. 1 *vs Okenia* sp. 2	17.4–17.9	10.1
*Okenia* sp. 1 *vs Okenia angelensis*	17.2–17.8	9.8
*Okenia* sp. 1 *vs Okenia zoobotryon*	17.9–18.3	10.1
*Okenia* sp. 2 *vs Okenia cupella*	-	11.6
*Okenia zoobotryon vs Okenia angelensis*	12.9	3.1
*Okenia picoensis vs Okenia felis*	16.6	-
*Okenia amoenula vs Okenia mediterranea*	5.9	0.9
*Okenia aspersa vs Okenia elegans*	13.2	3.7

### Systematics

Order Nudibranchia Cuvier, 1817

Family Goniodorididae H. Adams & A. Adams, 1854

**Genus *Okenia* Menke, 1830**

Type species *Idalia elegans* Leuckart, 1828 by monotypy

For a detailed synonymy and diagnosis of the genus see Rudman [4].

***Okenia longiductis* sp. nov**.

(formerly *Okenia* sp. 1)

(Figs 3–6)

**Fig 3:**
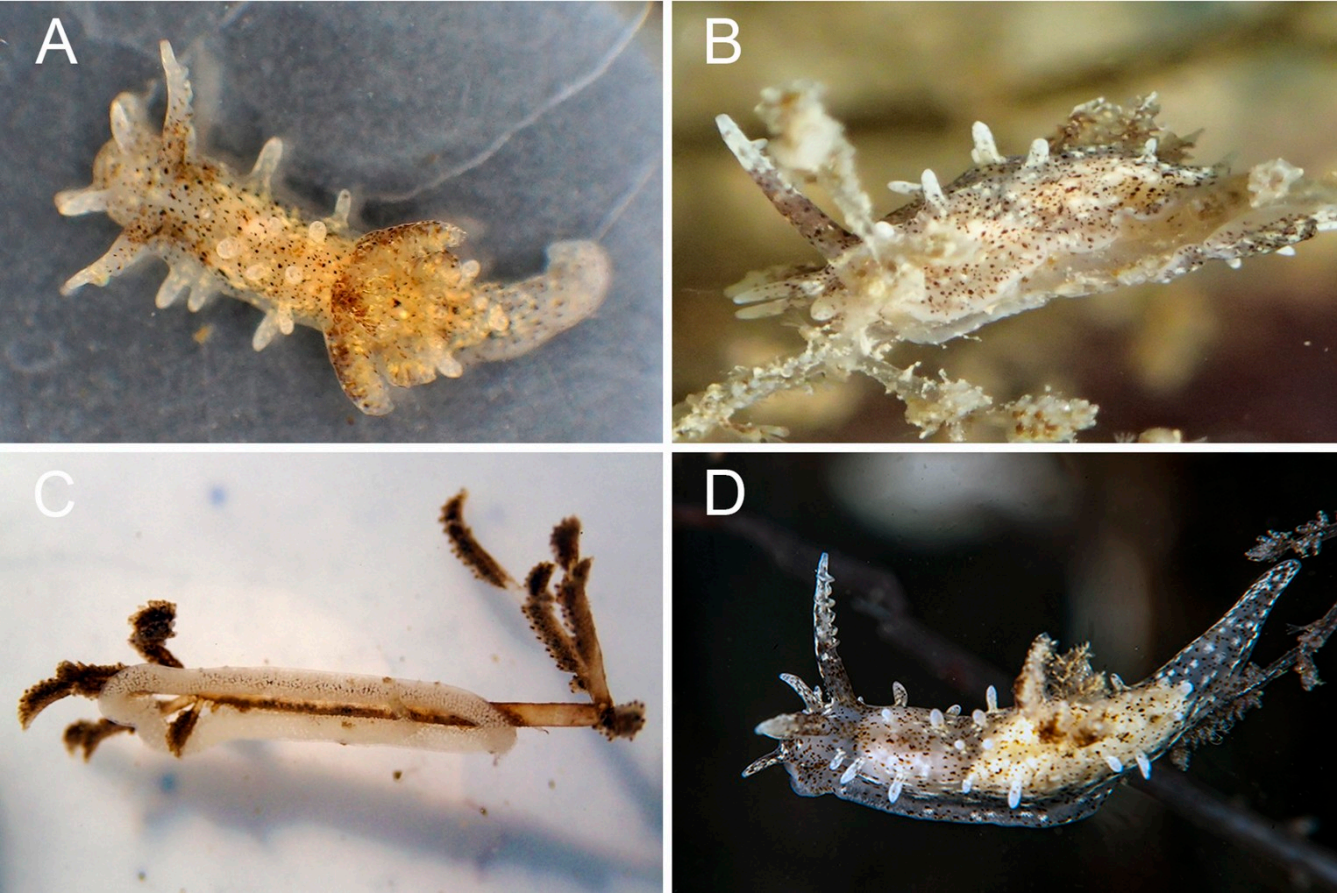
*Okenia longiductis* sp. nov. A. Specimen from Lago di Sabaudia (Italy). Photograph by A. Macali. B. Specimen from La Grande-Motte (France). Photograph by D. Minchin. C. Egg-masses on Amathia verticillata from La Grande-Motte (France). Photograph by D. Minchin. D. Specimen from Mar Piccolo, Taranto (Italy). Photograph by G. Colucci. Size (alcohol-preserved specimens) ~9 mm maximum length.

**Fig 4:**
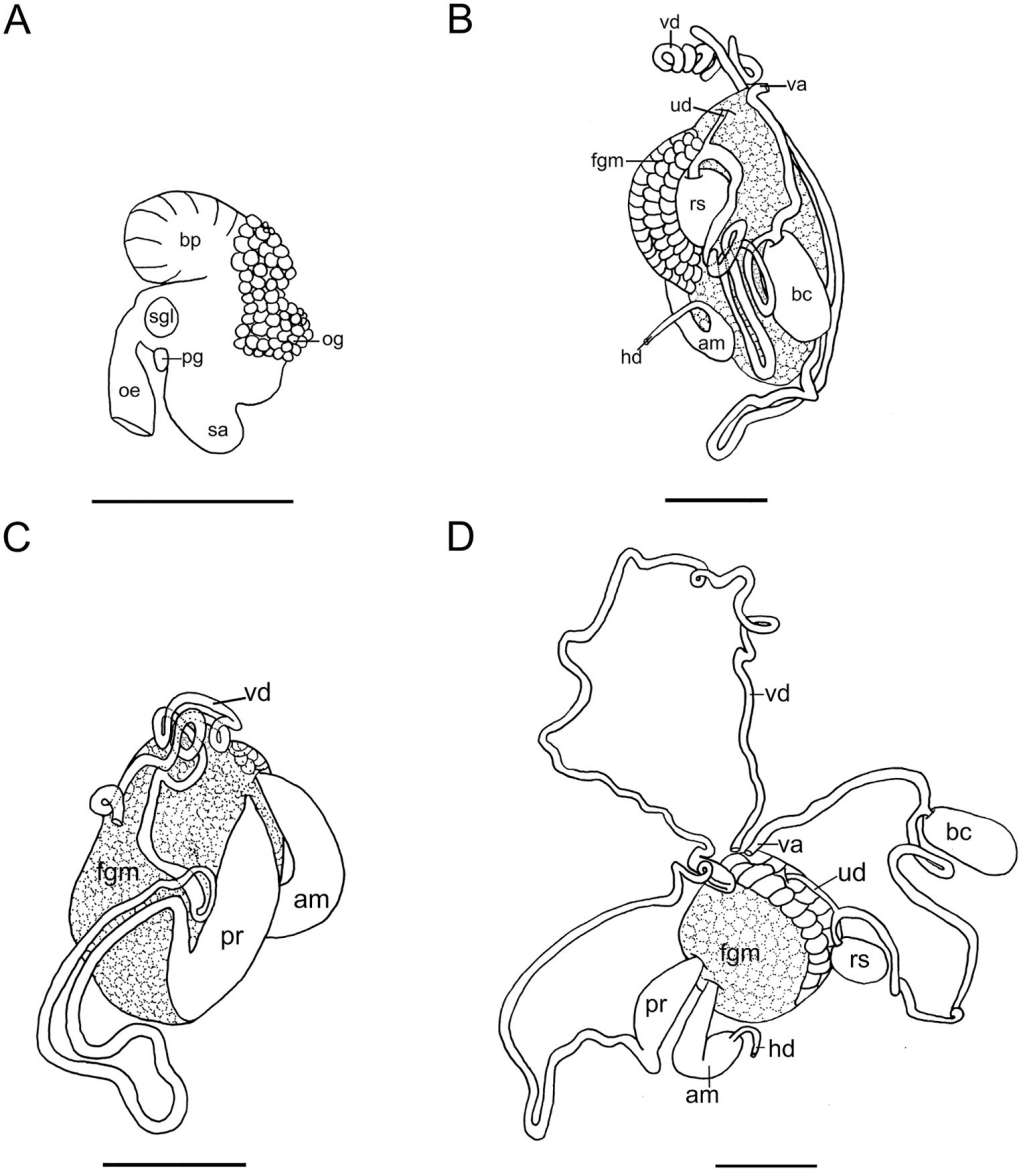
*Okenia longiductis* sp. nov. Internal anatomy. A. Buccal bulb. B. Female portion of the reproductive system. C. Male portion of the reproductive system. D. Reproductive system extended. Abbreviations: am, ampulla; bc, bursa copulatrix; bp, buccal pump; fgm, female gland mass; hd, hermaphroditic duct; oe, oesophagus; og, oral glands; pr, prostate; ra, radular sac; rs, receptaculum seminis; sgl, salivary gland; ud, uterine duct; va, vagina; vd, vas deferens. Scale bars: 1 mm.

**Fig 5:**
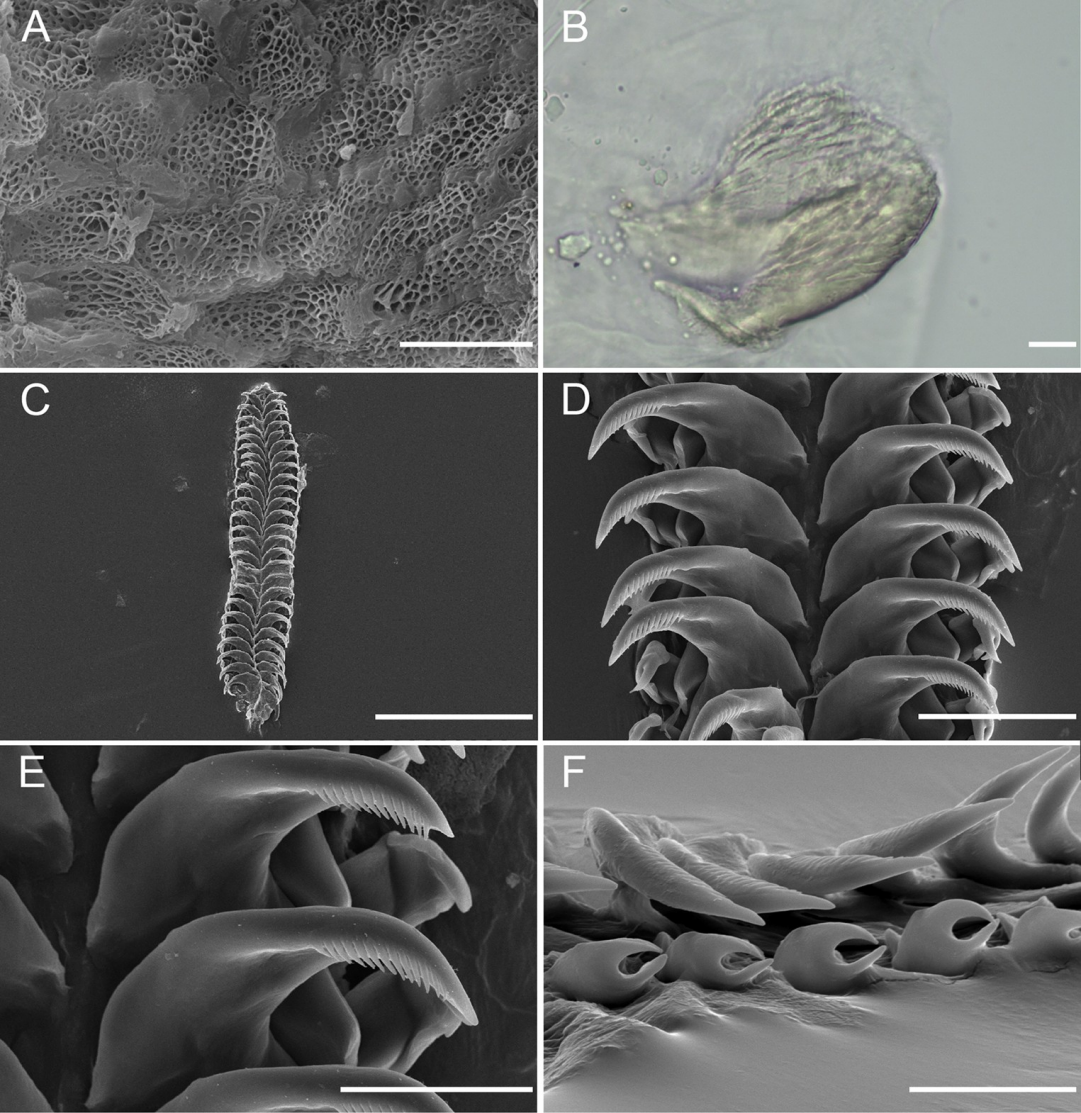
*Okenia longiductis* sp. nov. Scanning electron micrographs (SEM) and light microscope photographs (LMP). A. SEM. Detail of jaw elements (SZN-MOL0019). B. LMP. Detail of cuticle elements surrounding lips (SZN-MOL0006). C. SEM. Frontal view of entire radula (MNCN15.05/88169). D. SEM. Frontal view of radula. Detail of rachis, internal, and external teeth (MNCN15.05/88172). E. SEM. Detail of internal teeth (MNCN15.05/88172). F. SEM. Detail of external teeth (MNCN15.05/200036). Scale bars: A, 10 μm; B, 10 μm; C, 300 μm; D, 50 μm; E, 30 μm; F, 30 μm.

**Fig 6:**
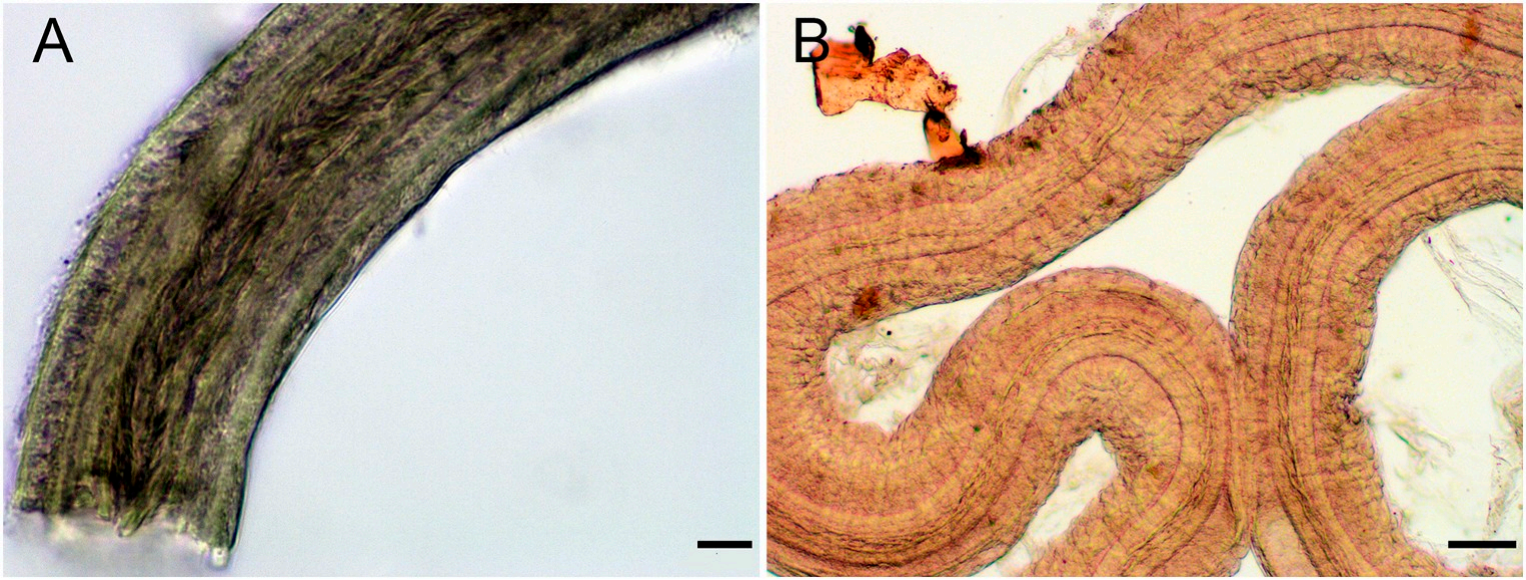
*Okenia longiductis* sp. nov. Light microscope photographs (LMP). A. Distal part of the penis, with penial spines (MNCN15.05/200040). B. Vas deferent with penial spines (SZN-MOL0005). Scale bars: 1mm.

LSID urn:lsid:zoobank.org:act:C74C4087-4BC6-48E9-8819-BAABDB3BAFB7

*Okenia zoobotryon sensu* Trainito & Doneddu [74]: 23 (Fig).

*Okenia* cf. *zoobotryon sensu* Ballesteros et al. [54]: 8.

*Okenia zoobotryon sensu* Lipej et al. [101]: 134–135 (Figs).

#### Type material

*Holotype*: Lago di Sabaudia (Italy), 0–1 m (Table 2), 3 mm preserved (dissected) (Table 3) (MNCN15.05/200035). *Paratypes* (P): Lago di Sabaudia (Italy), 0–1 m (Table 2; Fig 3A): P1–3 mm preserved (dissected) (Table 3) (SZN-MOL0003); P2–3.5 mm preserved (dissected) (Table 3) (SZN-MOL0004); P3–3 mm preserved (dissected) (Table 3) (MNCN15.05/70411); P4–4 mm preserved (dissected) (Table 3; Fig 5F) (MNCN15.05/200036). Mar Piccolo (Italy), 3–5 m (Table 2; Fig 3D): P5–9 mm preserved (dissected) (Table 3; Fig 6B) (SZN-MOL0005); P6–7 mm preserved (dissected) (Table 3) (MNCN15.05/200037). Porto Ercole (Italy), 0–1 m (Table 2): P7–7.8 mm alive, 5 mm preserved (dissected) (Table 3; Fig 5B) (SZN-MOL0006); P8–5.8 mm alive (Table 3) (MNCN15.05/200038); P9–5.4 mm alive (Table 3) (SZN-MOL0007); P10–6.2 mm alive (Table 3) (MNCN15.05/200039). Naples (Italy), 0–1 m (Table 2): P11–7.8 mm alive (dissected) (Table 3; Fig 6A) (MNCN15.05/200040); P12–7 mm alive (Table 3) (SZN-MOL0008); P13–6.1 mm alive (Table 3) (MNCN15.05/200041); P14–5.4 mm alive (Table 3) (SZN-MOL0009); P15–8.9 mm alive (Table 3) (MNCN15.05/200042).

#### Other material

Lago di Sabaudia (Italy), 0–1 m (Table 2; Fig 3A): 5 mm preserved (dissected) (SZN-MOL0011); 5 mm preserved (dissected) (SZN-MOL0010); 4 mm preserved (dissected) (MNCN15.05/88164); 7 mm preserved (MNCN15.05/88165). Mar Piccolo (Italy), 3–5 m (Table 2; Fig 3D): 7 mm preserved (dissected) (MNCN15.05/88166); 6 mm preserved (SZN-MOL0012); 8 mm preserved (Table 3) (SZN-MOL0013); 6 mm preserved (SZN-MOL0014); 6 mm preserved (MNCN15.05/88167); 6 mm preserved (MNCN15.05/88168). Porto Ercole (Italy), 0–1 m (Table 2): 7 mm alive (Table 3) (SZN-MOL0015); 6.8 mm alive (dissected) (Table 3; Fig 5C) (MNCN15.05/88169); 6.1 mm alive (dissected) (Table 3) (MNCN15.05/88170). Naples (Italy), 0–1 m (Table 2): 6.8 mm alive (Table 3; Fig 5A) (SZN-MOL0019); 5.8 mm alive (Table 3) (SZN-MOL0017); 8 mm alive (Table 3) (SZN-MOL0018); 6.2 mm alive (Table 3) (MNCN15.05/88171). La Grande-Motte (France), 0–0.5 m (Table 2; Fig 3B): 3 mm preserved (dissected) (SZN-MOL0016); 6 mm preserved (dissected) (Fig 5D) (MNCN15.05/88172); 3 mm preserved (MNCN15.05/88173).

#### Etymology

Named longiductis due to its long reproductive ducts.

#### External morphology (Fig 3)

Preserved specimens up to 9 mm maximum length. Elongated body ending in long and pointed posterior end of foot. Well-developed notal border with variable range of lateral and dorsal papillae, always symmetrically distributed on each body side. Lateral papillae: 5–8 on each body side; 1 additional papilla may be present in most posterior part of notum. Distribution as follows: 2 located in front of rhinophores, 1 behind gill, 3–6 between rhinophores and gill. Lateral papillae elongated, relatively short and thin. Dorsal papillae: 5–11. Distribution as follows: at least 3 papillae always present in front of gills and 1 behind rhinophores; remaining papillae (1–7) dispersed. Shape of dorsal papillae may vary, being similar to laterals or small bumps in mantle. Rhinophores long and slender, bearing 4–6 lamellae each. Gill composed of 7–8 tripinnate branches surrounding anus. Two large and well-developed oral tentacles, one each side of mouth. Foot elongate, rounded at anterior part, without visible propodial tentacles. Reproductive opening on right lateral side of body, located in first third of body. Entire body covered by conspicuous spicules.

#### Colour pattern (Fig 3)

Translucent white background, slightly grey due to transparency of internal organs; body with scattered white, dark brown, light brown, and cream spots. Dark brown spots more concentrated around base of rhinophores, outer face of gill branches and mouth; in remaining parts, intensity of brown spots variable among different specimens, sometimes giving a general brown tone. Papillae with white spots and few random brown spots. Translucent posterior end of foot with scattered white and brown dots, similar to body. Translucent foot with few dispersed dark brown spots.

#### Foregut anatomy

Buccal bulb thick and muscular (Fig 4A). Large number of rounded oral glands (og) surround anterior opening of bulb (Fig 4A). Buccal pump (bp) large and expanding dorsally and backwards (Fig 4A). Radular sac (ra) short, descending ventrally (Fig 4A). Thin oesophagus (oe) inserting into buccal bulb behind buccal pump (Fig 4A). One rounded salivary gland (sgl) on both sides of oesophagus (Fig 4A). Labial cuticle surrounding lips and expanding inside buccal pump; thin and weak part located inside buccal bulb with honeycomb-shaped jaw elements (Fig 5A); harder elements surrounding lips (Fig 5B). Radular formula of all dissected specimens 26–30×1.1.0.1.1 (Fig 5C). Shape of teeth similar in all localities. Inner lateral tooth with single large and robust cusp and robust and wide base (Fig 5D–5E). Cusp large, robust, and pointed with internal masticatory margin usually bearing 10–13 fine, pointed denticles; centrals being longer than those located at the ends (Fig 5D–5E). Posterior end of cusp well developed, ending in a sort of prominent wing (Fig 5D–5E). Outer lateral tooth much smaller, with large base and 2 relatively thin and pointed cusps, upper one wider than lower one (Fig 5F).

#### Reproductive system

Large reproductive system located in anterior third of body. Hermaphroditic duct (hd) thin and long beginning in ovotestis, located inside digestive-hermaphroditic gland, then expanding into a kidney-shaped ampulla (am) (Fig 4B–4D). Postampullatory duct narrow, connecting ampulla to female gland mass (fgm), dividing into oviduct and swollen prostatic portion (pr) of vas deferens (vd) (Fig 4C–4D). Prostate becomes narrow again, continues as an extraordinarily long and coiled duct, especially in its most distal part, where there are many closed loops (Fig 4B–4D). Vas deferens long, widening approximately in middle part and continuing narrowing towards thin ejaculatory duct, ending in penis (Fig 4C–4D). Penis with penial spines (Fig 6). Spines elongate and covering much of vas deferens (Fig 6). Thickness of vagina (va) similar to ejaculatory end of vas deferens (Fig 4D). Vagina considerably long connecting with big and elongated bursa copulatrix (bc) (Fig 4B and 4D). Base of the bursa copulatrix connected to the receptaculum seminis (rs) by a very long, thin, and finely coiled duct (Fig 4B and 4D). Receptaculum seminis slightly smaller and rounder than the bursa copulatrix (Fig 4B and 4D). Thin uterine duct (ud) entering female gland and emerging at base of receptaculum seminis (Fig 4B and 4D). Female gland very well developed (Fig 4B–4D).

#### Distribution

Mar Piccolo (Taranto), Naples, Lago di Sabaudia (Latina), Porto Ercole (Grosseto) (Italy), and La Grande-Motte (Hérault) (France) (present study). Literature records of *O*. *zoobotryon* from the Mediterranean Sea [54, 74, 101] (Fig 1; Table 1) also belong to this taxon. In fact, despite we were not able to study morphologically or molecularly those specimens as they were only mentioned in species lists or have disappeared, present evidences strongly point towards repetitive misidentification in the Mediterranean Sea, and thus we listed them here in the synonymy of the newly described species.

#### Ecology

We always found this species living in the infralittoral zone (up to 5 m depth) on the arborescent bryozoan *Amathia verticillata* (delle Chiaje, 1822) (Fig 3B and 3D). The same depths and feeding association hold for previous records belonging to *Okenia zoobotryon* [54, 74, 101]. White and ring-shaped egg-masses were present on this bryozoan (Fig 3C).

#### Remarks

*Okenia longiductis* sp. nov. resembles *Okenia zoobotryon* (Smallwood, 1910) and *Okenia angelensis* Lance, 1966 due to their similar general body shape and external colour pattern. *Okenia zoobotryon* is a taxon originally described from Bermuda [99–100] and recently reported from some Mediterranean localities—Pialassa Baiona, Ravenna (Italy), 23.09.2012: Trainito & Doneddu [74], F. Ioni, pers. comm.; Cala Maset, Sant Feliu de Guíxols (Spain): Ballesteros et al. [54]; entire coastline of Slovenia: Lipej et al. [101]. *Okenia angelensis* is only known to occur in a wide area of the Eastern Pacific [108–110]. However, our morphological analyses revealed clear external and internal differences between *O*. *longiductis* sp. nov. and these other taxa. A detailed comparison between these three taxa is shown in Table 5. Moreover, our molecular results, including those from species delimitation analyses, support that the three taxa are valid and distinct species (Fig 2), with a COI uncorrected *p*-distance of 17.2–17.8% between *O*. *longiductis* sp. nov. and *O*. *angelensis* and of 17.9–18.3% between *O*. *longiductis* sp. nov. and *O*. *zoobotryon* (Table 4).

**Table 5:** Differences between *Okenia longiductis* sp. nov., *Okenia angelensis*, and *Okenia zoobotryon*. Data after Smallwood [9,9], Pola [100], Lance [10,8], Gosliner & Bertsch [3], and present study.

	*Okenia longiductis* sp. nov.	*Okenia angelensis*	*Okenia zoobotryon*
**External anatomy**:			
**general colour pattern**	translucent body with scattered white, light and dark brown, and creamy spots; white spots and few random brown spots on papillae	white translucent body with yellow and white spots; white spots on papillae	white hyaline body with scattered brown spots; white spots on papillae
**dorsal papillae**	5–11, elongated	6–9, elongated	4, elongated
**gill branches**	7–8	5–7	4–6
**lamellae**	4–6	1–3	3–6
**Internal anatomy**:			
**ducts lenght**	very long	medium	medium
**ampulla**	kidney-shaped	sausage-shaped	short-oval
**penial spines**	present	absent	absent
**bursa copulatrix**	large and elongate	spherical	spherical

***Okenia problematica* sp. nov**.

(formerly *Okenia* sp. 2)

(Figs 7–9)

**Fig 7:**
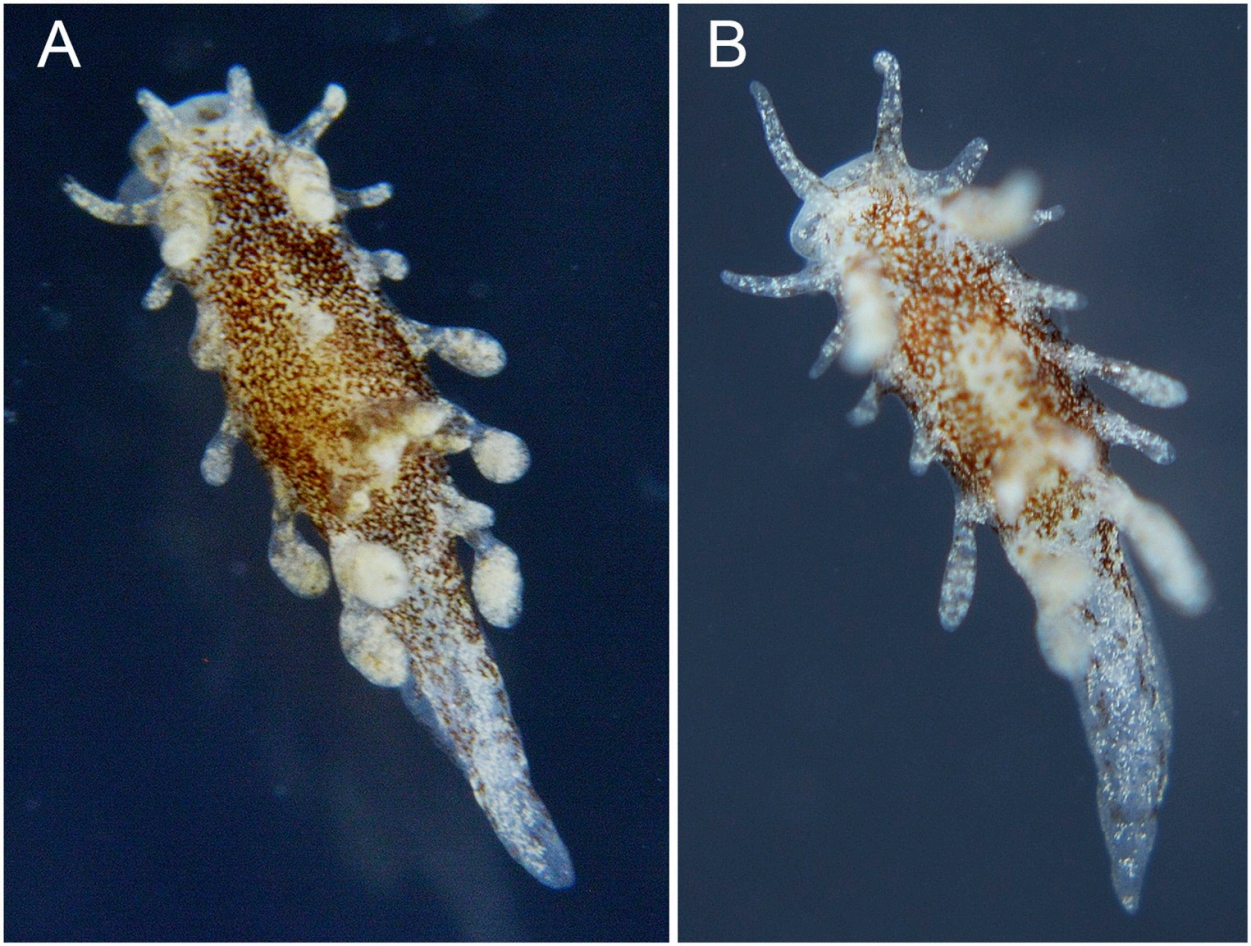
*Okenia problematica* sp. nov. Living animals from Gallipoli (Italy). A. Holotype (MNCN15.05/200034); B. Paratype (SZN-MOL0001). Photographs by F. Vitale. Size (alcohol-preserved specimens) ~2.5 mm maximum length.

**Fig 8:**
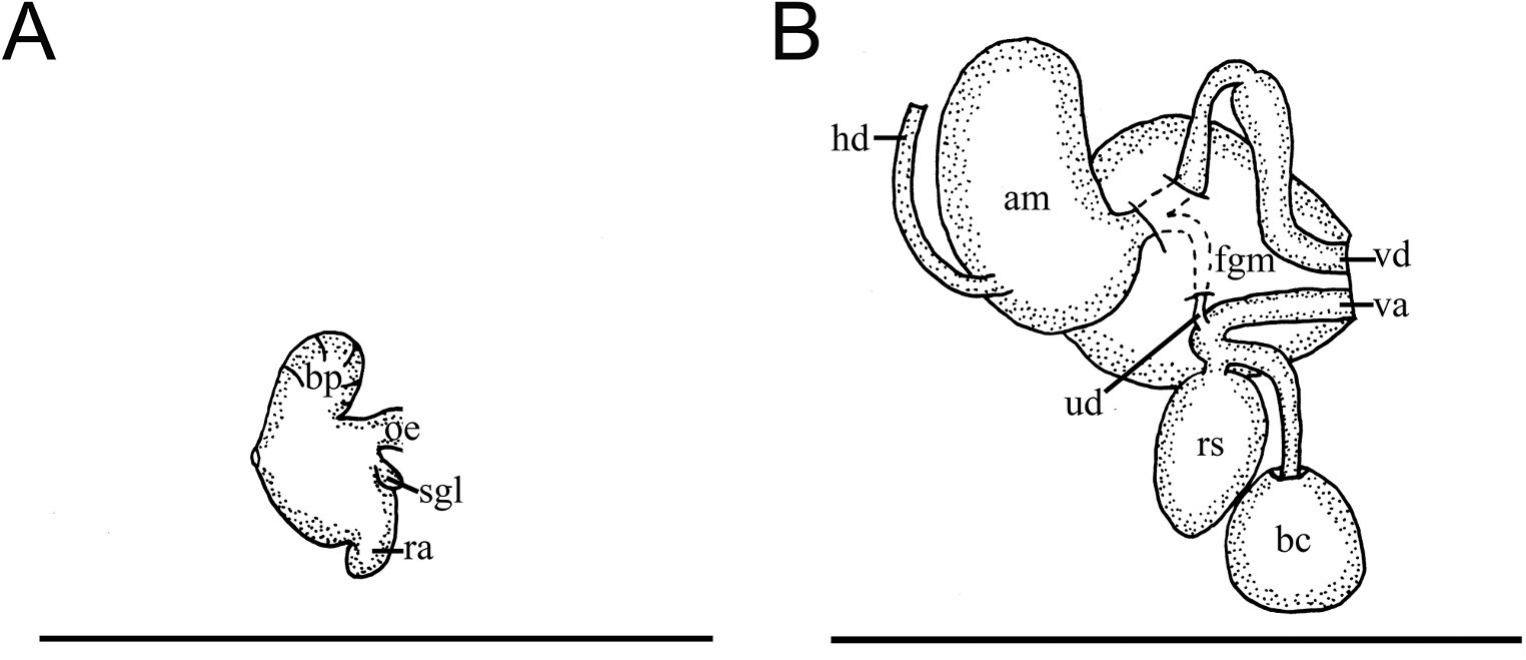
*Okenia problematica* sp. nov. Internal anatomy. A. Buccal bulb. B. Reproductive system. Abbreviations: am, ampulla; bc, bursa copulatrix; bp, buccal pump; fgm, female gland mass; hd, hermaphroditic duct; oe, oesophagus; ra, radular sac; rs, receptaculum seminis; sgl, salivary gland; ud, uterine duct; va, vagina; vd, vas deferens. Scale bars: 1 mm.

**Fig 9:**
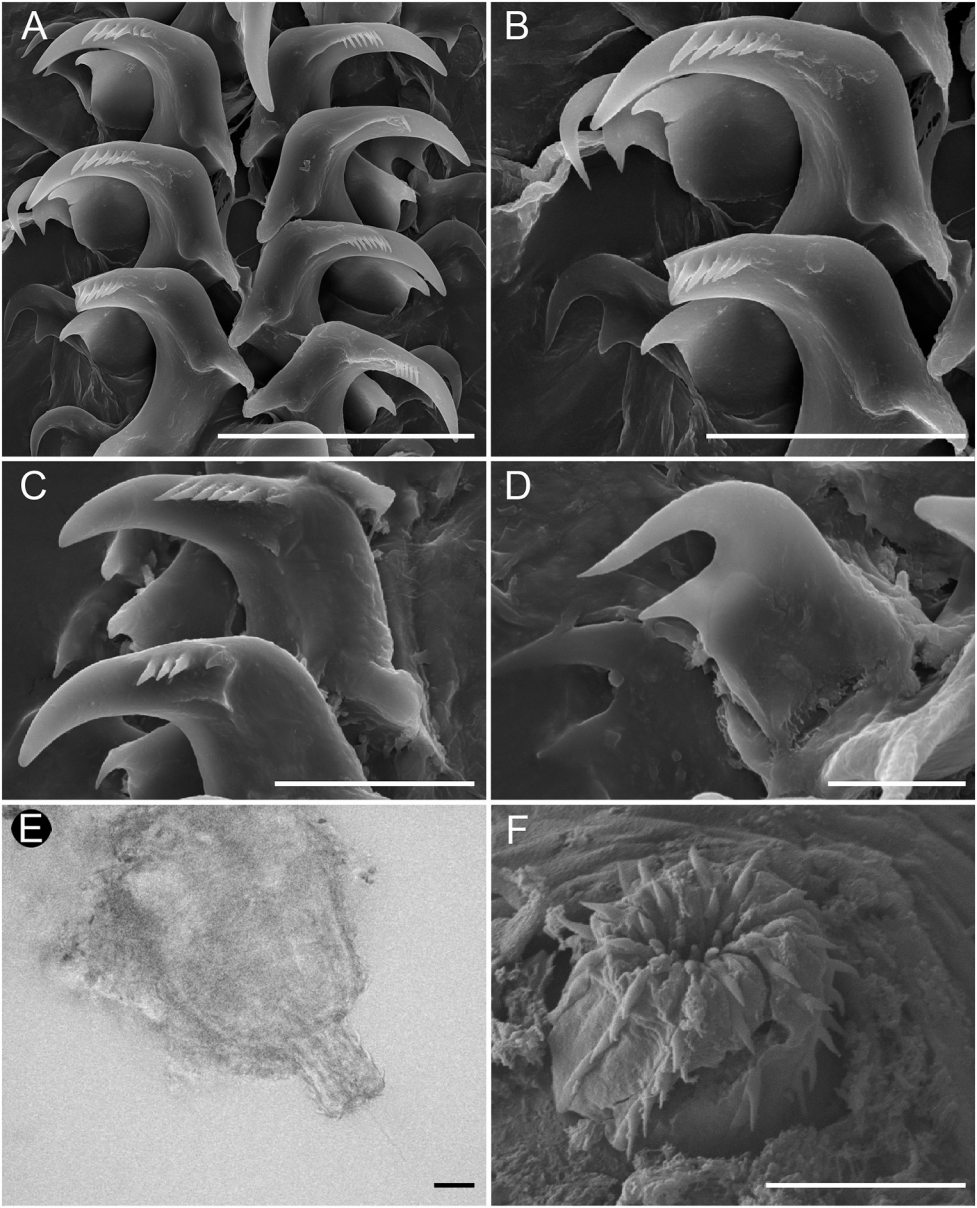
*Okenia problematica* sp. nov. Scanning electron micrographs (SEM) and light microscope photographs (LMP). A. SEM. Frontal view of radula. Detail of rachis, internal, and external teeth (MNCN15.05/200034). B. SEM. Detail of internal and external lateral teeth (MNCN15.05/200034). C. SEM. Detail of internal teeth (SZN-MOL0002). D. SEM. Detail of external tooth (SZN-MOL0002). E. LMF. Detail of penial spines (MNCN15.05/200034). F. SEM. Detail of penial spines (SZN-MOL0002). Scale bars: A, 50 μm; B, 30 μm; C, 20 μm; D, 10 μm; E, 10 μm; F, 20 μm.

LSID urn:lsid:zoobank.org:act:20FD7CCC-9C36-4604-BFB4-56AD468AF211

*Okenia impexa* Marcus, 1957 *sensu* Schmekel [57]: 355–360 (Figs 1 and 4–5).

*Okenia impexa* Marcus, 1957 *sensu* Schmekel & Portmann [85]: 126–128, 370–371 (Fig 9), 388–389 (Fig 7).

*Okenia impexa* Marcus, 1957 *sensu* Templado [98]: 250.

*Okenia cupella* (Vogel & Schultz, 1970) *sensu* Valdés & Ortea [18]: 230–231 (Fig 6).

*Okenia cupella sensu* Templado et al. [95]: 100, 197.

*Okenia cupella* (Vogel y Schultz, 1970) *sensu* García-Gómez et al. [52]: 464 (Fig).

*Okenia* cf. *impexa* Er. Marcus, 1957 *sensu* Ballesteros et al. [54]: 7–8 (Fig 2B).

#### Type material

*Holotype*: Gallipoli (Italy), 30 m (Table 2): 2 mm preserved (dissected) (Table 3; Figs 7A, 8, 9A–9B and 9E) (MNCN15.05/200034). *Paratype*: Gallipoli (Italy), 30 m (Table 2): (Fig 7B), 1.5 mm preserved (dissected) (Table 3) (SZN-MOL0001).

#### Other material

Aiguafreda (Spain), 10 m (Table 2): 2.5 mm preserved (dissected) (Fig 9C–9D and 9F) (SZN-MOL0002); 2 mm preserved (dissected) (MNCN15.05/88162). Cala Joncols (Spain), 11 m (Table 2): 2 mm preserved (MNCN15.05/88163).

#### Etymology

Named problematica due to its complex taxonomic history.

#### External morphology (Fig 7)

Preserved specimens up to 2.5 mm maximum length. Elongated body ending in long and pointed posterior end of foot. Well-developed notal border with 8 lateral papillae symmetrically distributed on each body side. Distribution of papillae as follows: 1 located in front of rhinophores, 1 at same level of rhinophores, 4 between rhinophores and gill, 2 behind gill, both arising from same stalk. Shape and size of papillae slightly variable; anteriormost 2 papillae on each side long and finger-like, followed by 1 shorter and subsequently increasing in size. All papillae behind rhinophores with rounded widening at tip, more or less evident, being more marked in papillae behind gill. Single papilla in mid-dorsal line similar in size and shape to lateral ones, also rounded tipped. Rhinophores long and slender, bearing 6–9 lamellae each. Gill composed of 4 unipinnate branches surrounding anus. Two anteriormost branches sharing stalk. Two short oral tentacles, one each side of mouth. Foot long and slender, with 2 small but elongated propodial tentacles in anterior part. Reproductive opening on right lateral side of body, usually at short distance from rhinophore. Entire body covered by conspicuous spicules.

#### Colour pattern (Fig 7)

White translucent background; body and gill covered with many and concentrated brown spots; white-yellowish spots scattered randomly. Rhinophores and papillae translucent with cream-white and scattered light brown spots. White translucent tail with mostly white and scattered brown dots. White translucent foot without spots.

#### Foregut anatomy

Buccal bulb thick and muscular (Fig 8A). Labial glands absent. Buccal pump (bp) large and expanding dorsally and backwards (Fig 8A). Radular sac (ra) short descending ventrally (Fig 8A). Thin oesophagus (oe) inserting into buccal bulb behind buccal pump (Fig 8A). One small and rounded salivary gland (sgl) on both sides of oesophagus (Fig 8A). Labial cuticle surrounding lips and expanding inside buccal pump. Small jaw elements present, but lost in all samples during manipulation before SEM. Radular formula of Gallipoli (Italy) specimens 12×1.1.0.1.1. Spanish specimens with similar formula (10–12×1.1.0.1.1). Shape of teeth similar in both localities (Fig 9B–9C). Inner lateral tooth large and hook-shaped with strong base (Fig 9B–9C). Upper cusp large, robust, and pointed with internal masticatory margin usually bearing 7–9 thin long denticles, decreasing in size progressively from tip to lower part of tooth (Fig 9B–9C). Posterior end of cusp well developed, ending in a sort of prominent wing (Fig 9A–9C). Lower cusp smaller, curved, and pointed (Fig 9A–9C). Outer lateral tooth even smaller, with large base and 2 relatively thin and pointed cusps, upper one longer than lower one (Fig 9D).

#### Reproductive system

Reproductive system located in anterior third of body. Hermaphroditic duct (hd) elongated and beginning in ovotestis, located inside digestive-hermaphroditic gland, then expanding into a large and kidney-shaped ampulla (am) (Fig 8B). Postampullatory duct short, connecting ampulla to female gland (fgm), dividing into oviduct and prostatic portion of vas deferens (vd). Prostate not morphologically differentiated. Vas deferens widens in middle part and continues slightly narrowing towards ejaculatory duct, ending in penis. Penis with thin and relatively long and hooked penial spines (Fig 9E–9F). Thickness of vagina (va) similar to ejaculatory end of vas deferens (Fig 8B). Vagina relatively long connecting with rounded bursa copulatrix (bc) (Fig 8B). Receptaculum seminis (rs) pear-shaped arising in middle of vagina, supported by short duct (Fig 8B). Thin uterine duct (ud) entering female gland and emerging in base of receptaculum seminis (Fig 8B).

#### Distribution

Gallipoli (Lecce, Italy), Aiguafreda (Barcelona, Spain), and Cala Joncols (Girona, Spain) (present study). Literature records of *Okenia cupella* and *Okenia impexa* from the Mediterranean Sea [18, 52, 54, 57, 85, 95, 98] (Fig 1; Table 1), except that of *Okenia pusilla* Sordi, 1974 from Ischia Island [96] (see below in Remarks), also belong to this taxon. In fact, despite attemps to examine previously collected samples, we were not able to study morphologically or molecularly those specimens as they were only mentioned in species lists or have disappeared from collections. However, we present evidence strongly pointing towards repetitive misidentification in the Mediterranean Sea, and thus we listed them here in the synonymy of the newly described species.

#### Ecology

We always found *Okenia problematica* sp. nov. at depths below 10 m. The specimens collected in Gallipoli (Italy) were found on an artificial reef located on a sandy bottom, amidst unidentified hydrozoans and encrusting bryozoans. No environmental data were collected for the Spanish specimens (Aiguafreda and Cala Joncols). Templado [98], Valdés & Ortea [18], García-Gómez et al. [52], and Ballesteros et al. [54] mostly reported its cryptic presence in rocky infralittoral bottoms (~5–10 m) with the bryozoan *Margaretta cereoides* (Ellis & Solander, 1786). However, Schmekel [57], Schmekel & Portmann [85], Valdés & Ortea [18], and Ballesteros et al. [54] also reported its presence at 5–15 m depth on infralittoral algae such as *Halimeda* Lamouroux or Corallinaceae Lamouroux taxa and *Codium vermilara* (Olivi) Delle Chiaje.

#### Remarks

*Okenia problematica* sp. nov. has had a troublesome taxonomic history. In fact, it is very likely to have been already recorded in the Mediterranean Sea both as *Okenia cupella* (Vogel & Schultz, 1970) and as *Okenia impexa* Er. Marcus, 1957. Moreover, two names related to *O*. *cupella* and *O*. *impexa* were introduced based on Mediterranean material, namely *Okenia pusilla* Sordi, 1974 and *Okenia impexa banyulensis* Schmekel, 1979 (Table 1). Such a nomenclatural chaos presumably comes from the extreme similarities of this group of species with a similar external colour pattern and characteristic tip of the papillae, as well as the absence of detailed descriptions of holotypes and/or topotypical specimens. *Okenia cupella* was originally described from the western Atlantic, in Virginia [94]. Despite its great similarities in external appearance and radular features with other *Okenia* species, this taxon has been widely recorded from both sides of the Atlantic Ocean [12, 51], including the western Mediterranean Sea (Table 1; Fig 1). The situation is similar for *O*. *impexa*, originally described from Brazil [97], recorded from both sides of the Atlantic Ocean [111–112] and also from the western Mediterranean Sea (Table 1; Fig 1). However, we found that our specimens from the Mediterranean supposedly belonging to either taxa, actually belong to the same entity. To clarify the taxonomic position of our specimens we morphologically and molecularly studied the holotype of *O*. *cupella* (see redescription below and Table 3), whereas for *O*. *impexa* we had to rely on the original description [97] and the topotypical specimens figured by Sales et al. [113], as the type material only consists of slides of syntypes [113]. Our morphological analyses based on published and new data revealed several major differences between *O*. *problematica* sp. nov. and *O*. *impexa* and *O*. *cupella* (Table 6). Moreover, the molecular results for the H3 gene analysis confirm the observed morphological dissimilarities, supporting the hypothesis that *O*. *problematica* sp. nov. and *O*. *cupella* are different species, with a H3 uncorrected *p*-distance of 11.6%, value higher than other H3 uncorrected *p*-distances within and between species (Table 4).

**Table 6:** Differences between *Okenia problematica* sp. nov., *Okenia cupella*, and *Okenia impexa*. Data after Marcus [9,7], Vogel & Schultz [9,4], Sales et al. [11,3], and present study.

	*Okenia problematica* sp. nov.	*Okenia cupella*	*Okenia impexa*
**External anatomy**:			
**general colour pattern**	translucent-white body with brown spots densely concentrated along notum and lateral sides of body, giving a general brown appearance; translucent papillae mostly with creamy-white spots	whitish body with brown spots mainly located between rhinophores and mid-dorsal papillae	whitish body with a dense brown net, forming denser patches on back, yellow anterior border of head; tips of dorsal papillae yellow
**posterior lateral papillae**	rounded	rounded	claviform
**Internal anatomy**:			
** cusps in the lateral teeth**	2	2	3
**vas deferens**	widening in middle part and lacking a differentiate prostatic portion	no widening in middle part and wider prostatic portion of vas deferens	no widening in middle part and wider prostatic portion of vas deferens
**duct in connection with receptaculum seminis**	short, arising from middle of vagina	short, arising from near bursa copulatrix	long, arising from near bursa copulatrix
**receptaculum seminis**	large	small	large
**bursa copulatrix**	large and rounded	small and rounded	large and elongated

The identity of the material that provided the basis for the names *O*. *pusilla* and *O*. *impexa banyulensis* is also uncertain. *Okenia pusilla* was originally described based on the external anatomy and radular features of a single specimen deposited at CIBM—Centro Interuniversitario di Biologia Marina ed Ecologia Applicata “G. Bacci” (Livorno, Italy). Sordi [96] described this species as having four brown gill branches, with the two anteriormost sharing the same stalk, eight papillae on each side of the body, yet lacking dorsal papillae, a character highlighted in the original description in order to differentiate between *O*. *pusilla* and *O*. *impexa*—see also the holotype drawn in Sordi [96] and photographed in Cattaneo-Vietti et al. [71]. However, our specimens always bore dorsal papillae, despite being of similar size with respect to the holotype of *O*. *pusilla*. Furthermore, the poor description of the radular features did not enable us to compare our material with that described by Sordi [96]. Unfortunately, it was not possible to retrieve the type material for a direct comparison, as this material has been presumably lost (S. De Ranieri, pers. comm.). For these reasons, *O*. *pusilla* cannot be ascribed with confidence to any known *Okenia* species, including *O*. *problematica* sp. nov. For the sake of nomenclatural stability, we here state that *O*. *pusilla* should be recognized as a *nomen dubium* (ICZN [106]: glossary).

Schmekel [57] described *Okenia impexa banyulensis* based on two main differences when compared with the original description of *O*. *impexa*: *i*) the shape of the lateral tooth, with two cusps in the Mediterranean specimens *vs* three cusps in the western Atlantic specimens; *ii*) the shape of papillae, with Mediterranean specimens having very short, pointed, digitiform tubercles and lateral posterior appendages of the notum border apically swollen and rounded *vs* club-shaped tubercles and claviform though pointed lateral posterior appendages in western Atlantic specimens. Indeed, specimens recorded by Schmekel [57] would appear to be conspecific with *O*. *problematica* sp. nov. Again, our efforts to locate the material analyzed by Schmekel [57] failed, as it is presumably lost. In fact, it was not found in the Natural History Museum Basel (Switzerland) nor the Natural History Museum of Munich (Germany) (Michael Schrödl, pers. comm.), where it was originally deposited [57]. Furthermore, Schmekel [57] wrote “If further observations in the western Atlantic confirm the constancy of the above mentioned geographical differences, I name the eastern Atlantic subspecies *Okenia impexa banyulensis*”. Thus, having been published after 1960 and proposed conditionally, *O*. *impexa banyulensis* is not available under ICZN [106] rules (art. 15.1) (see also Table 1).

*Okenia cupella* (Vogel & Schultz, 1970)

(Figs 10–12)

**Fig 10:**
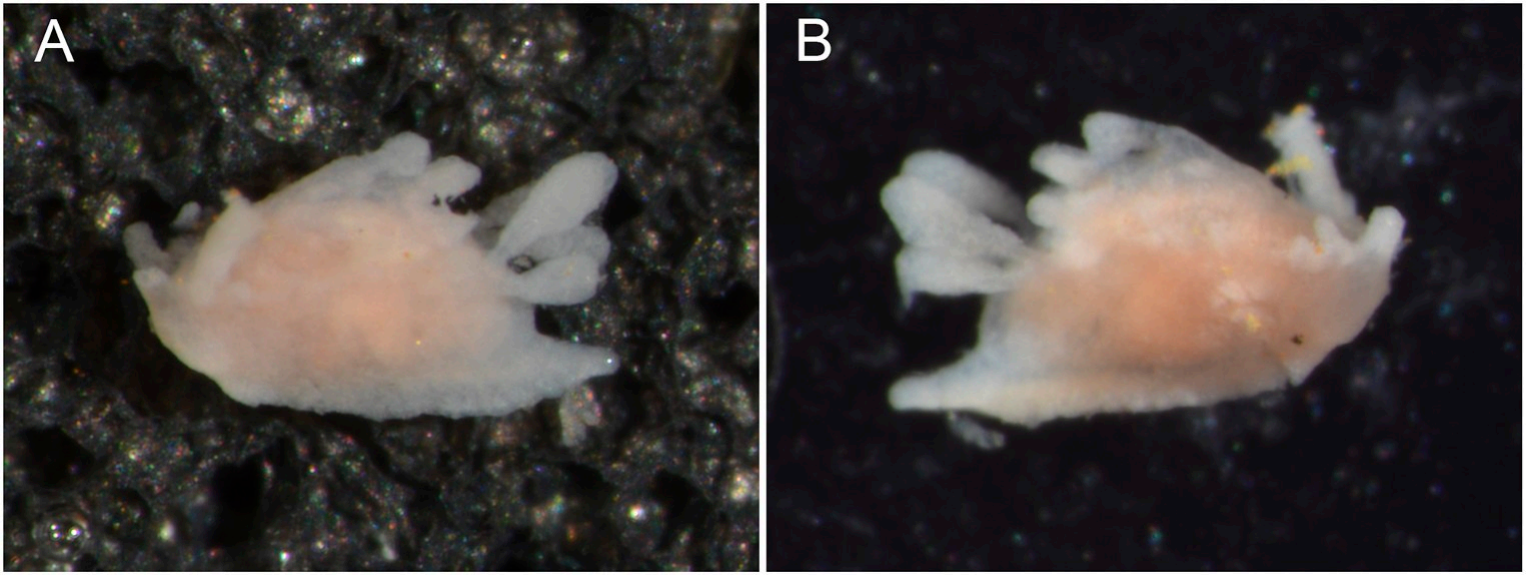
*Okenia cupella* (holotype, USNM 679396). Lateral views of the specimen.

**Fig 11:**
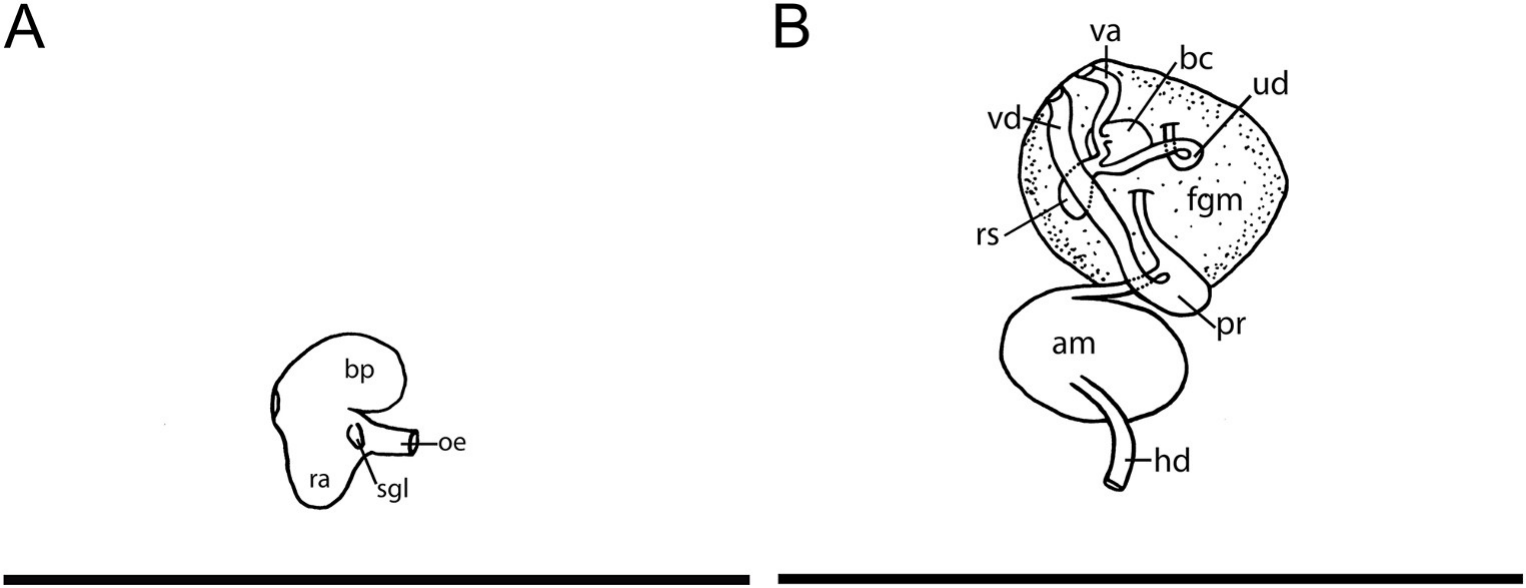
*Okenia cupella* (holotype, USNM 679396). Internal anatomy. A. Buccal bulb. B. Reproductive system. Abbreviations: am, ampulla; bc, bursa copulatrix; bp, buccal pump; fgm, female gland mass; hd, hermaphroditic duct; oe, oesophagus; ra, radular sac; rs, receptaculum seminis; sgl, salivary gland; ud, uterine duct; va, vagina; vd, vas deferens. Scale bars: 1 mm.

**Fig 12:**
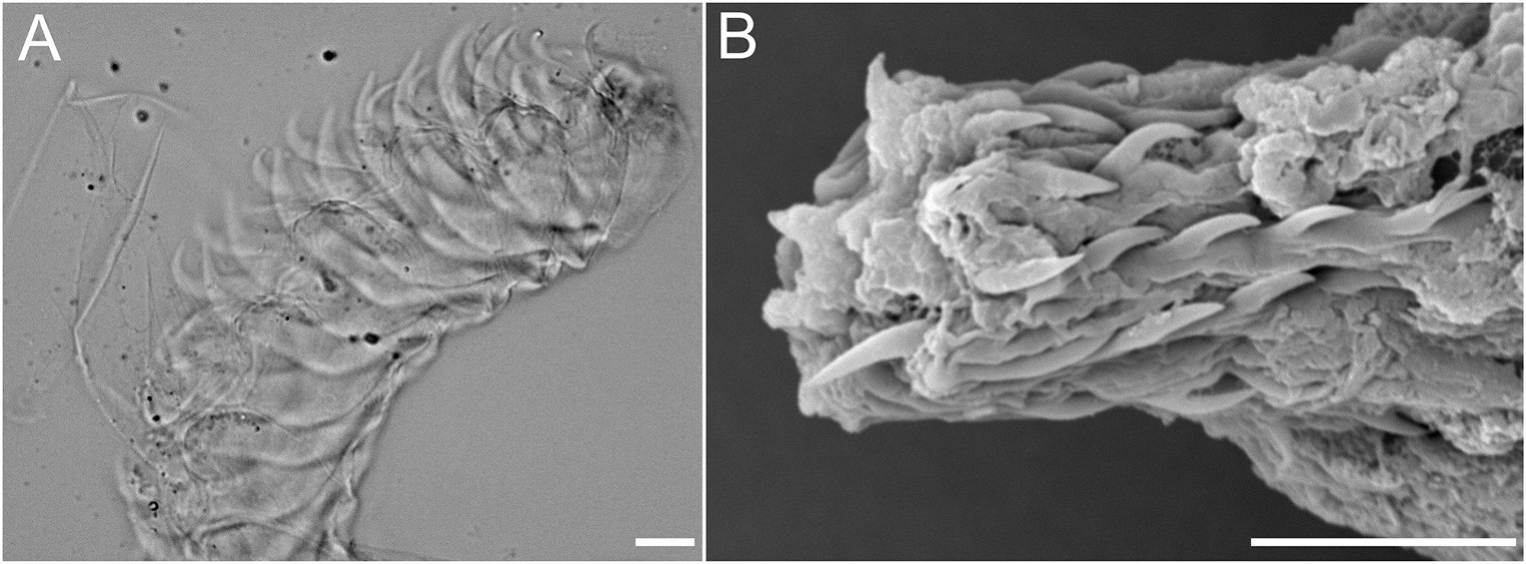
*Okenia cupella* (holotype, USNM 679396). Scanning electron micrographs (SEM) and light microscope photographs (LMP). A. LMF. Radula. B. SEM. Detail of penial spines. Scale bars: 10 μm.

#### Material examined

Holotype. Aberdeen Rock, York River (Virginia, U.S.A.), *legit* David G. Cargo, 01.11.1968: 1 spm (Figs 10–12) (USNM 679396).

#### External morphology

Preserved specimen not reaching 1 mm maximum length (Fig 10). Original description of external morphology is complete and well detailed (see Vogel & Schultz [94]; Figs 1–3), and therefore we avoided including it here.

#### Foregut anatomy

Buccal bulb thick and muscular (Fig 11A). Buccal pump (bp) large and rounded, expanding dorsally and backwards (Fig 11A). Radular sac (ra) descending ventrally (Fig 11A). Thin oesophagus (oe) inserting into buccal bulb behind buccal pump, this union being surrounded by nervous system. Small and slightly elongated salivary glands (sgl) on either side of oesophagus (Fig 11A). Labial cuticle surrounding lips and expanding inside buccal pump. Radular formula observed under light microscope: 10–11×1.1.0.1.1 (Fig 12A), thus fitting its original description: radula bearing 10 rows of teeth pointed posteriorly, and inner lateral teeth with masticatory margin with 9 denticles [90]. Due to tiny size and transparency of radula, we were not able to extend it properly in order to prepare and examine it in detail with scanning electron microscopy.

#### Reproductive system

Reproductive system located in anterior part of body. Thin and elongate hermaphroditic duct (hd) beginning in ovotestis, located inside digestive-hermaphrodite gland, then expanding into big, thick, and rounded ampulla (am), being almost half of entire reproductive system (Fig 11B). Postampullatory duct thin, connecting ampulla to female gland (fgm), dividing into thin oviduct and slightly wider prostatic portion of vas deferens (vd) (Fig 11B). Distal end of prostatic part continues as a long and wide ejaculatory duct, ending in penis. Penis with large, wide, and hooked penial spines (Fig 12B). Vagina (va) shorter and thinner than vas deferens, connecting with rounded bursa copulatrix (bc). Receptaculum seminis (rs) elongate arising near to this union. Receptaculum seminis slightly smaller than bursa copulatrix. Long and once rolled uterine duct (ud) entering female gland and emerging in base of receptaculum seminis.

## Discussion

The taxonomy and biogeography of *Okenia* species in the Mediterranean Sea has been mainly based on external morphology, with the sole exception of very few works which studied the internal anatomy of selected specimens [17, 18, 57], and the majority of papers dealing with local biota relied only on external resemblances for identification (Table 1 and references therein). However, despite the presence of diagnostic characters even in external morphology (e.g. general colour and shape of papillae), identifications of *Okenia* taxa have proved to be a challenging task worldwide, resulting in multiple misidentifications that have confused the geographical distribution of species within this genus. This indeed asked for the necessity of an in-depth morphological and molecular review of Mediterranean *Okenia* species.

**Fig 13:**
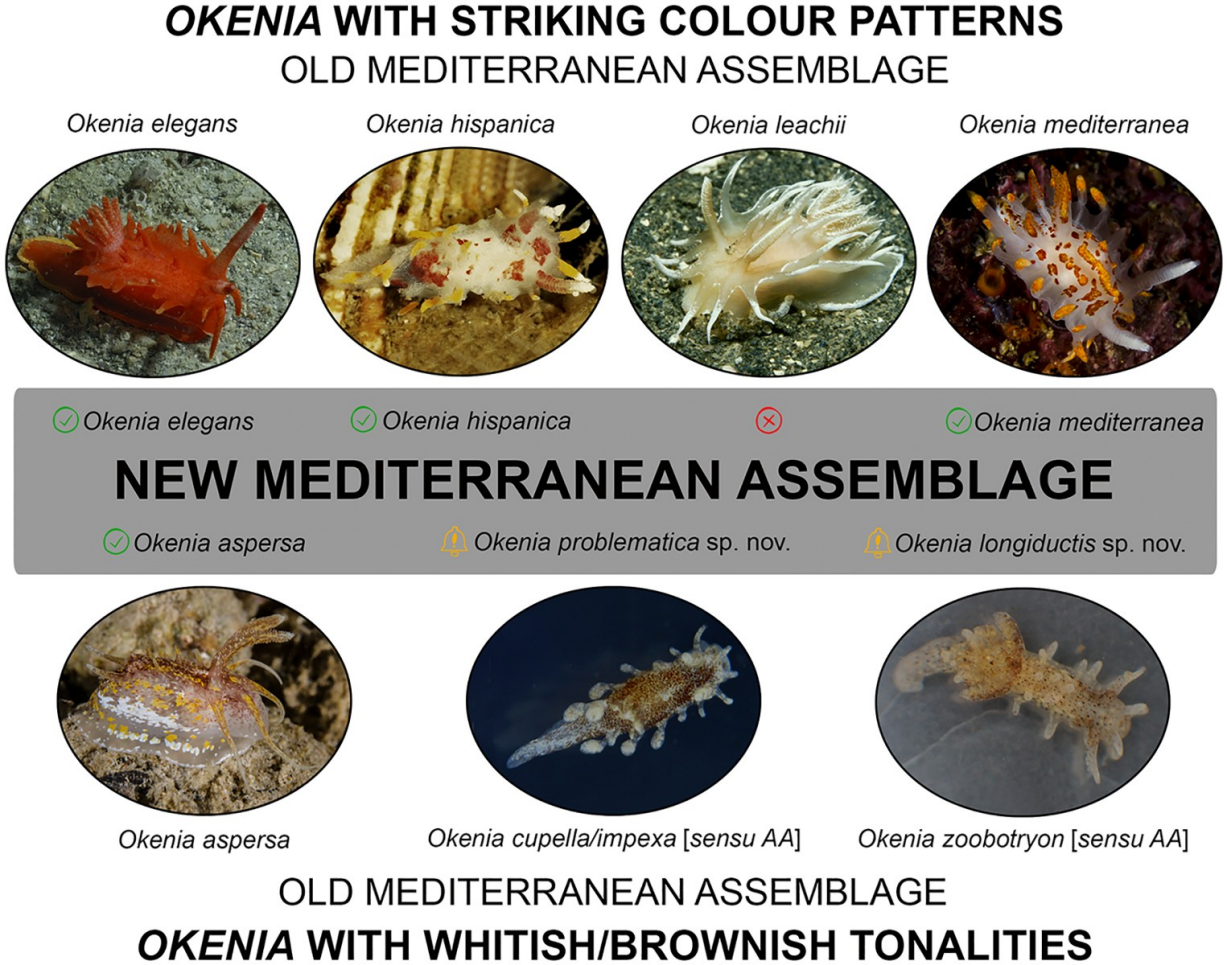
Old and new Mediterranean *Okenia* assemblage. *Okenia elegans*. Miramare (Trieste, Italy) [65–66]. Photo: D. Poloniato. *Okenia hispanica*. Holotype. Estepona (Malaga, Spain) [52, 74]. Photo: D. Moreno/Fauna Ibérica (MNCN-CSIC). *Okenia leachii*. Eilean Siar (Great Britain) [74]. Photo: B. Picton. *Okenia mediterranea*. Santa Maria al Bagno (Lecce, Italy) (Table 2: MNCN15.05/88174). Photo: F. Vitale. *Okenia aspersa*. Noli (Savona, Italy) [19, 91]. Photo: F. Betti. *Okenia problematica* sp. nov. (= *Okenia cupella/impexa sensu* AA [18, 52, 54, 57, 85, 95, 98]). Gallipoli (Italy) (Table 4: MNCN15.05/200034). Photo: F. Vitale. *Okenia longiductis* sp. nov. (= *Okenia zoobotryon sensu* AA [54, 74, 101]). Lago di Sabaudia (Italy) (Table 2). Photo: A. Macali.

Based on our preliminary literature research, eight valid species were usually reported from the Mediterranean Sea, namely *Okenia aspersa* (Alder & Hancock, 1845), *Okenia cupella* (Vogel & Schultz, 1970), *Okenia elegans* (Leuckart, 1828), *Okenia hispanica* Valdés & Ortea, 1995, *Okenia impexa* Er. Marcus, 1957, *Okenia leachii* (Alder & Hancock, 1854), *Okenia mediterranea* (Ihering, 1886), and *Okenia zoobotryon* (Smallwood, 1910). However, not all of them can be considered to be actually occurring in the area (Fig 13). This result should not be considered surprising as many molluscan groups of the Mediterranean Sea have not been fully subjected to a focused review based on literature and modern genetic methods, and Mediterranean checklists are often still being compiled. In addition, deletion of species reported in local, national, or Mediterranean checklists is an ongoing and time-consuming process necessary towards a homogenization of the general knowledge of the Mediterranean malacofauna [31, 114–120].

Interestingly, two of the six remaining species, *O*. *cupella/impexa* [18, 52, 54, 57, 85, 95, 98] and *O*. *zoobotryon* [54, 74, 101], were constantly misidentified in the past literature. We name here the specimens previously identified as those species for the Mediterranean Sea as *Okenia problematica* sp. nov. and *Okenia longiductis* sp. nov., respectively. Our result agrees with the current molluscan literature for the Mediterranean Sea. Indeed, the validity of many taxa described or recorded from the Mediterranean Sea over centuries has yet to be confirmed by molecular means or by barcoding of material [21]. The study of selected phylogenetic clades, through integrative approaches, has already brought to unexpected results, including the discovery or description of several new species even within widely studied groups [24, 27–29, 121–122]. At the same time, our result also confuted the possible occurrence of *O*. *zoobotryon* in the Mediterranean Sea, thus contributing to shed light on local bioinvasions.

Unfortunately, the elusive character of *Okenia* taxa has prevented us to answer additional questions that still remain unresolved. We were unable to obtain fresh material of *O*. *aspersa*, and we relied on a specimen previously barcoded from nearby its Atlantic type locality. Several authors have already highlighted differences between Mediterranean and eastern Atlantic specimens [2, 16–18, 85]. Consequently, should cryptic diversity be eventually found within this taxon, no name is available for Mediterranean specimens, taking also into account that *Doris quadricornis* Montagu, 1815 [= *Okenia quadricornis* (Montagu, 1815)] was suppressed in 1974 under ICZN Opinion 1014 [102], and thus it will again result in a species new to science. Another Mediterranean species worth a mention is *O*. *hispanica*. Despite being described more than 20 years ago, it is still only known from its holotype [18, 52, 74, 123] (Fig 13). The presence of Mediterranean endemisms, with a narrow distribution restricted to the Alborán Sea, is already a well-known phenomenon [124–125], although debated in the recent literature [126]. Taking into account that no freshly collected specimens were available for this study, and that the holotype was originally fixed in formaldehyde, further field work is necessary to determine if *O*. *hispanica* is a truly Mediterranean endemism or the knowledge of its distribution is still incomplete; new collections are also required to evaluate its phylogenetic relationships with congeneric taxa. Finally, the external and internal anatomy of the *O*. *mediterranea* specimen studied here did fit well with that previously described by Cervera et al. [17]. However, Schmekel [57] and Cervera et al. [17] already highlighted that *O*. *mediterranea* is made up of two different morphs highly variable in their external anatomy and colour pattern. Unfortunately, the original description lacks detail of its internal anatomy, and therefore a topotype is needed to evaluate putative taxonomic differences between these colour morphs.

In summary, the present study significantly clarifies the biodiversity of the genus *Okenia* in the Mediterranean Sea, by *i*) recapitulating all the known records of species within this genus, and highlighting the questionable ones; *ii*) restricting the taxonomically validated records to *O*. *aspersa*, *O*. *elegans*, *O*. *hispanica*, and *O*. *mediterranea*, although leaving doubts on some of those taxa; *iii*) adding *O*. *problematica* sp. nov and *O*. *longiductis* sp. nov to the present Mediterranean biota; *iv*) resolving the status of some intricated taxonomic problems, and proposing *Okenia pusilla* as a “*nomen dubium*”. Finally, it restricts the presence of *O*. *cupella*, *O*. *impexa*, and *O*. *zoobotryon* to the western Atlantic, and of *O*. *leachii* to the eastern Atlantic. A comparative table highlighting main external diagnostic characters and radular formula of *Okenia* species living in the Mediterranean is reported in Table 7.

**Table 7:** Comparative table highlighting main external diagnostic characters and radular formula of *Okenia* species living in the Mediterranean Sea.

	*Okenia* *aspersa*	*Okenia* *elegans*	*Okenia* *hispanica*	*Okenia longiductis* sp. nov.	*Okenia mediterranea*	*Okenia problematica* sp. nov.
**general colour pattern**	translucent body, mostly marked by yellowish, reddish brown, and orange blotches; white sprinkled sides; fawn rhinophores, brown and white freckled; pale fawn gill branches, speckled with darker shade and large white patch near apices	very variable: body generally light red, white, or pinkish; white and yellow spots can be present; papillae generally yellow, orange, or purple, sometimes with red or yellow spots; rhinophores and gill branches pinkish or reddish with yellow tips; yellow-orange edge of notum and foot	hyaline white body with pink patches, two largest ones behind each rhinophore; large pink patch on the anal area; yellow papillae with white apex; white gill branches	translucent body with scattered white, light and dark brown, and creamy spots; dark brown spots concentrated around base of rhinophores, outer face of gill branches and mouth; papillae mostly with white spots and few brown ones	white body with three yellow lines above notum; red spots inside yellow markings are present in one of the two known colour forms; rhinophores, papillae, and gill branches with white, yellow, or red spots	translucent-white body with several brown spots and few scattered white-yellowish spots; rhinophores and papillae translucent with creamy-white spots, some brown scattered spots present
**lateral papillae**	6–8	14–19	9	5–8	8–12	7–8
**dorsal papillae**	none	4–6	-	5–11	4–12 tubercles	1
**gill branches**	10–12	17–22	5	7–8	5–9	4
**lamellae**	43	70–80	?	4–6	12–20	6–9
**radular formula**	26×1.1.0.1.1	33–35×1.1.0.1.1	20×1.1.0.1.1	26–30×1.1.0.1.1	25×1.1.0.1.1	10–12×1.1.0.1.1
